# Cost-Effectiveness of Acceptable-Quality Deceased Donor Kidneys for Transplant in Older Candidates

**DOI:** 10.1001/jamanetworkopen.2025.55428

**Published:** 2026-01-27

**Authors:** Matthew B. Kaufmann, Jane C. Tan, Douglas K. Owens, Glenn M. Chertow, Jeremy D. Goldhaber-Fiebert

**Affiliations:** 1 Division of Health Policy and Management, University of Minnesota, Minneapolis; 2 Department of Health Policy, Stanford University School of Medicine, Stanford, California; 3 Center for Health Policy, Freeman Spogli Institute, Stanford University, Stanford, California; 4 Department of Medicine, Stanford University School of Medicine, Stanford, California

## Abstract

**Importance:**

Many acceptable-quality deceased donor kidneys go unused every year. Older transplant candidates are more vulnerable to rapid health decline.

**Objective:**

To assess the cost-effectiveness of increasing the kidney transplantation rate among older patients with end-stage kidney disease by using acceptable-quality deceased donor kidneys.

**Design, Setting, and Participants:**

This economic evaluation with cost-effectiveness analysis uses a microsimulation model of the kidney transplantation process for a synthetic cohort of adult candidates aged 65 years or older on the transplant waiting list between 2010 and 2019 over their remaining lifetimes. Statistical analysis was performed from January 2023 to December 2025.

**Intervention:**

Increasing the transplantation rate in 5% increments higher than the status quo rate from 5% to 25% using acceptable-quality deceased donor kidneys, with a corresponding shift in the distribution of kidney quality to reflect the use of donor kidneys of lower quality than the status quo.

**Main Outcomes and Measures:**

The primary outcomes were the number of key waiting list and posttransplant outcomes, costs, quality-adjusted life-years (QALYs), and incremental cost-effectiveness ratios. Health state transition probabilities were derived from Scientific Registry of Transplant Recipients data. Costs and quality-of-life weights were derived from published literature and US Renal Data System annual reports, all of which were varied in a probabilistic sensitivity analysis.

**Results:**

In this synthetic cohort of 100 000 individuals, the mean age was 68.8 years (95% CI, 65.0-78.0 years), they had received dialysis for a mean of 1.2 years (95% CI, 0-6.6 years), 61.7% were male, and 56.8% had diabetes. It was estimated there would be 141 (range, 118-161) fewer waiting list deaths per 10 000 candidates if the deceased donor transplantation rate were increased by 25%. Increasing the deceased donor transplantation rate by 25% would cost $8100 (95% credible interval, $700-$14 100) per QALY gained or was cost saving from the health care sector and modified health care sector perspectives. From the health care sector perspective, a 25% increase in the deceased donor transplantation rate was the preferred strategy in all probabilistic sensitivity analysis samples for willingness-to-pay thresholds of $40 000 or more per QALY gained.

**Conclusions and Relevance:**

This economic analysis of increasing the kidney transplantation rate in older adults suggests that using acceptable-quality deceased donor kidneys would be cost-effective or cost saving. Decision-makers should consider policies that make better use of recovered kidneys to increase transplantation rates among older patients and any other patients with similar preferences.

## Key Points

**Question:**

What is the association of increasing the deceased donor kidney transplantation rate among older candidates using acceptable-quality kidneys with health and economic outcomes?

**Findings:**

This economic evaluation using a microsimulation model of the deceased donor transplant process for older adults found that increasing the transplant rate by 25% was associated with improved health outcomes and cost $8100 per quality-adjusted life-year gained from the health care sector perspective. This process became cost saving when considering patient and caregiver time costs.

**Meaning:**

These findings suggest that policies aimed at increasing access to acceptable-quality donor kidneys could be associated with cost-effective improvement of the health of older transplant candidates.

## Teaser

This economic evaluation assesses the cost-effectiveness and health outcomes of increasing the kidney transplantation rate among patients aged 65 years or older with end-stage kidney disease by using acceptable-quality deceased donor kidneys.

## Introduction

The US kidney transplant system does not operate at optimal efficiency. There were 71 523 patients on the kidney transplant waiting list at the end of 2022, and of those who were listed 5 years ago, 48.7% had received a transplant.^1^ For many patients, longevity and quality of life would be enhanced by a kidney transplant in lieu of dialysis, a treatment that sustains life but rarely restores health. Despite the urgent need, approximately 1 in 4 recovered deceased donor kidneys was not used.^2^ The proportion of recovered but ultimately unused deceased donor kidneys has steadily increased from 5.1% in 1988 to 24.6% in 2021.^2,3^ An estimated 17 435 unused donor kidneys (62%) in the US from 2004 to 2014 would have been used in France’s more liberal organ allocation system.^4^ The French system has many differences from the US system, including less regulatory scrutiny and a prohibition on performing a biopsy on deceased donor kidneys before transplantation. Biopsies are prohibited because they are believed to add little information about the quality of the donor kidney while prolonging the time-sensitive allocation process.^5^

Older patients with end-stage kidney disease (ESKD) undergo transplants at markedly lower rates than their younger counterparts.^6^ The probability of being evaluated, waitlisted, and receiving a transplant decreases as patients age and accumulate comorbid conditions and complications associated with ESKD.^7^ In 2021, those aged 65 years or older represented 42.8% of all patients with ESKD but only 23.6% of transplant candidates and 20.6% of patients who received a kidney transplant. Older transplant candidates receiving dialysis might be willing to trade off receiving a “lower-quality” deceased donor kidney for a shorter waiting time.^8^ Although older candidates may have heterogeneous preferences, the current system makes it difficult to fully incorporate patient preferences into decision-making. Thus, systematically operationalizing patient choice into critical moments of decision-making in the modern-day clinical platform is challenging.

The intense focus on transplant performance metrics has fostered increasingly conservative practices at many transplant centers. Metrics for evaluating transplant centers have been at odds with increasing access to transplants. For example, transplant centers may have reservations about a disproportionate pool of candidates at higher risk (including older candidates) and concern about nonjudicious use of lower-quality donor kidneys due to worry about negatively affecting results and potentially threatening the viability of the transplant program itself.^9^ Metrics that include first-year patient and graft survival, waiting list mortality rates, and transplantation rates are biased against transplants for patients who are older and sicker. Transplant centers should be able to provide health-enhancing, high-quality, high-value care for all patients, not only those in the best of health with the lowest complication rates.

We hypothesized that more widespread use of deceased donor kidneys of lower but acceptable quality would be associated with improved outcomes for older patients with ESKD in a cost-effective manner. In support of this policy discussion, we performed a model-based, cost-effectiveness analysis evaluating the benefits, harms, effectiveness, and cost-effectiveness associated with increasing the transplantation rate among older candidates by using lower but acceptable-quality recovered deceased donor kidneys.

## Methods

### Model Description

**Figure 1.**
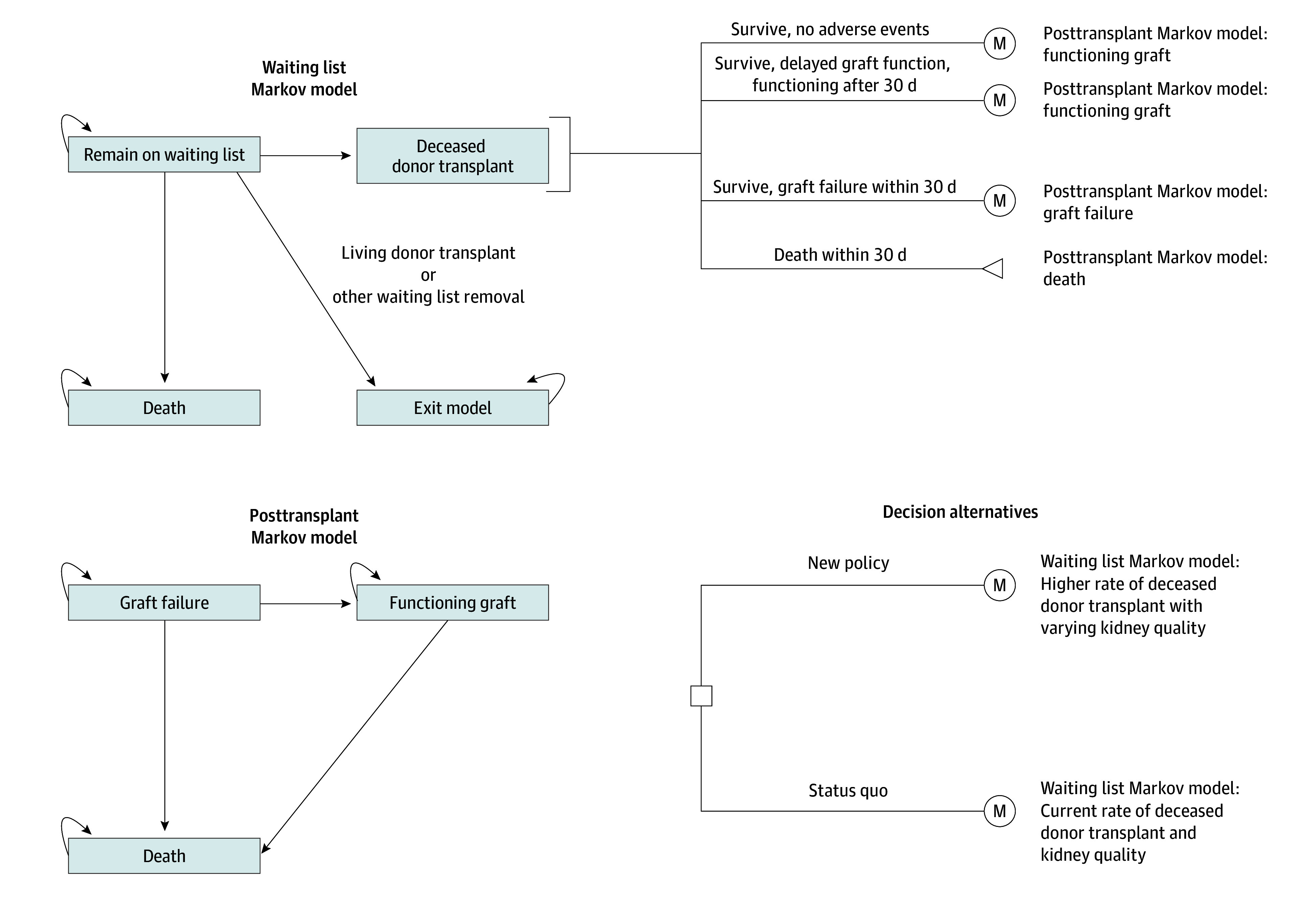
Model Diagram Reprinted with permission.^10^

We used a previously developed discrete time microsimulation model of the deceased donor kidney transplant process for older adults (aged ≥65 years) in the US.^10^ The model follows up with patients on the waiting list, tracking outcomes including deceased donor transplant, living donor transplant, waiting list mortality, and other reasons for waiting list removal. Other reasons for waiting list removal encompass removal for any reason other than death or transplant, including removal due to declining health. For those who receive a deceased donor kidney, the model then follows up with them for the rest of their lifetimes, starting with 30-day posttransplant, mutually exclusive outcomes: (1) no adverse events, (2) delayed graft function (DGF), (3) graft loss, or (4) death. We do not differentiate between graft loss and acute rejection, as acute rejection is a rare cause of graft loss in this population. Depending on their graft function and survival after the first 30 days, the model follows up with simulated patients over their remaining lives, tracking events including graft failure and death (Figure 1).^10^ We assumed that there is no chance for retransplantation. This study was exempt from institutional review board approval as it did not constitute human participant research. We adhered to the Consolidated Health Economic Evaluation Reporting Standards (CHEERS) reporting guideline.^11^

### Data and Sources

Transitions between health states depend on a series of risk equations that involve patient and donor characteristics. We previously estimated risk equations from data representing all US candidates and recipients who were waitlisted and underwent a transplant between 2010 and 2019.^12,13^ To account for competing risks, we used a likelihood-based model calibration to simultaneously match waiting list and transplant outcomes (eMethods 1, eTable 1, and eFigures 1-3 in Supplement 1). We updated the calibration using 64 equally weighted sets of calibrated parameters, which were all consistent with calibration targets (eFigures 1-3 and eTable 1 in Supplement 1).^10^

Kidney transplant care costs encompassing dialysis, transplant, and posttransplant events and complications were included in the analysis conducted from the health care sector perspective. We included Organ Acquisition Costs Center payments, which are sizable payments to transplant programs for transplant evaluation and waiting list management.^14^ Patient and caregiver time (opportunity) costs were included for our modified health care sector perspective analysis. We calculated caregiver time costs as the mean number of hours per month spent caregiving multiplied by mean wage estimates from the Bureau of Labor Statistics.^15,16,17^ We assumed that all transplant candidates have a caregiver. Caregiver costs and caregiver burden are lower after a transplant compared with while the patient is undergoing dialysis, but they are still substantial.^18^ We likewise calculated patient time costs for the time receiving care. The number of hours of caregiving and receiving care were generated through discussions with experts in transplant nephrology (Table 1).^14,15,16,17,19,20,21,22,23^ We assumed that older transplant candidates would have the same time costs as working-age patients. Excluding these costs would imply that their time has no value if they are not working. Additional information on cost calculations appear in the eMethods 2 and eTable 2 in Supplement 1.

**Table 1.** Model Inputs With Uncertainty Intervals

Parameter	Value (95% uncertainty interval) by age, y						Source
	65-69	70-79	75-79	80-84	≥85	All	
Health care sector perspective cost parameters, $							
Dialysis with Part D (monthly)	8791 (8520-9027)	8675 (8392-8975)	8553 (8261-8837)	8672 (8411-8956)	8387 (8077-8680)	NA	US Renal Data System,^19^ 2021
Posttransplant with Part D (monthly)	3520 (3365-3682)	3498 (3339-3663)	3604 (3293-3930)	3419 (3106-3727)	3182 (2880-3485)	NA	US Renal Data System,^19^ 2021
Deceased donor transplantation (1 time)	NA	NA	NA	NA	NA	114 687 (109 815-121 621)	Axelrod et al,^20^ 2017
Organ acquisition cost center (1 time)	NA	NA	NA	NA	NA	112 318 (56 338-186 604)	Cheng et al,^14^ 2022
Delayed graft function (1 time)	NA	NA	NA	NA	NA	41 592 (29 618-54 226)	Almond et al,^21^ 1991
Graft failure (1 time)	NA	NA	NA	NA	NA	85 989 (38 852-154 910)	US Renal Data System,^19^ 2021
Living donor transplant (1 time)	NA	NA	NA	NA	NA	111 976 (110 989-112 958)	Axelrod et al,^20^ 2017
Post–waiting list removal multiplier (range)^a^	NA	NA	NA	NA	NA	1.20 (1.00-1.40)	
Modified health care sector perspective cost parameters, $							
Caregiver time							
Dialysis (monthly)	NA	NA	NA	NA	NA	5867 (5655-6085)	US Bureau of Labor Statistics^15^; Liu et al,^16^ 2022
Posttransplant month 1 (monthly)^b^	NA	NA	NA	NA	NA	1355 (1097-1607)	US Bureau of Labor Statistics^15^; Langa et al,^17^ 2001
Posttransplant months 2-3 (monthly)^b^	NA	NA	NA	NA	NA	677 (548-803)	US Bureau of Labor Statistics^15^; Langa et al,^17^ 2001
Posttransplant months ≥4 (monthly)^b^	NA	NA	NA	NA	NA	276 (224-329)	US Bureau of Labor Statistics^15^; Langa et al,^17^ 2001
Patient time							
Dialysis (monthly)^b^	NA	NA	NA	NA	NA	1611 (1302-1919)	US Bureau of Labor Statistics^15^
Posttransplant month 1 (monthly)^b^	NA	NA	NA	NA	NA	372 (301-443)	US Bureau of Labor Statistics^15^
Posttransplant months 2-3 (monthly)^b^	NA	NA	NA	NA	NA	186 (150-221)	US Bureau of Labor Statistics^15^
Posttransplant months ≥4 (monthly)^b^	NA	NA	NA	NA	NA	62 (51-74)	US Bureau of Labor Statistics^15^
**Quality-of-life weights**							
Dialysis							
Male	0.46 (0.43-0.50)	0.42 (0.39-0.46)	0.42 (0.39-0.46)	0.40 (0.37-0.44)	0.40 (0.37-0.44)	NA	Wyld et al,^22^ 2012; Hanmer et al,^23^ 2006
Female	0.43 (0.39-0.47)	0.39 (0.36-0.43)	0.39 (0.36-0.43)	0.34 (0.31-0.38)	0.34 (0.31-0.38)	NA	Wyld et al,^22^ 2012; Hanmer et al,^23^ 2006
Posttransplant (0-3 mo)							
Male	0.58 (0.55-0.61)	0.54 (0.50-0.58)	0.54 (0.50-0.58)	0.52 (0.49-0.56)	0.52 (0.49-0.56)	NA	Wyld et al,^22^ 2012; Hanmer et al,^23^ 2006
Female	0.55 (0.51-0.59)	0.51 (0.47-0.55)	0.51 (0.47-0.55)	0.46 (0.43-0.50)	0.46 (0.43-0.50)	NA	Wyld et al,^22^ 2012; Hanmer et al,^23^ 2006
Posttransplant (4-8 mo)							
Male	0.66 (0.62-0.69)	0.63 (0.60-0.67)	0.63 (0.60-0.67)	0.61 (0.57-0.64)	0.61 (0.57-0.64)	NA	Wyld et al,^22^ 2012; Hanmer et al,^23^ 2006
Female	0.64 (0.60-0.67)	0.60 (0.56-0.64)	0.60 (0.56-0.64)	0.55 (0.51-0.59)	0.55 (0.51-0.59)	NA	Wyld et al,^22^ 2012; Hanmer et al,^23^ 2006
Posttransplant (9-12 mo)							
Male	0.60 (0.57-0.64)	0.56 (0.53-0.60)	0.56 (0.53-0.60)	0.54 (0.50-0.58)	0.54 (0.50-0.58)	NA	Wyld et al,^22^ 2012; Hanmer et al,^23^ 2006
Female	0.57 (0.53-0.61)	0.53 (0.49-0.57)	0.53 (0.49-0.57)	0.48 (0.45-0.52)	0.48 (0.45-0.52)	NA	Wyld et al,^22^ 2012; Hanmer et al,^23^ 2006
Posttransplant (≥13 mo)							
Male	0.59 (0.53-0.59)	0.55 (0.52-0.59)	0.55 (0.52-0.59)	0.53 (0.49-0.57)	0.53 (0.49-0.57)	NA	Wyld et al,^22^ 2012; Hanmer et al,^23^ 2006
Female	0.56 (0.53-0.59)	0.52 (0.49-0.56)	0.52 (0.49-0.56)	0.47 (0.43-0.51)	0.47 (0.43-0.51)	NA	Wyld et al,^22^ 2012; Hanmer et al,^23^ 2006
Delayed graft function (1 time)^a^	NA	NA	NA	NA	NA	0	
Graft failure (1 time)^a^	NA	NA	NA	NA	NA	0	

Abbreviation: NA, not applicable.

^a^ Assumed.

^b^ Expert opinion.

At the start of the simulation, 25.8% of patients had undergone dialysis for less than 1 month; we assumed all patients received dialysis going forward. A common nonfatal reason for waiting list removal is declining health rendering patients unable to receive a transplant. We therefore assumed that patients removed from the waiting list incurred additional health care costs that were 20% higher than the costs for patients who remained on the waiting list. We varied this value in our sensitivity analysis.

We derived health-related quality-of-life (HRQOL) weights that were age and sex specific after transplant as well as age, sex, and time-since-transplant specific after transplant (Table 1).^14,15,16,17,19,20,21,22,23^ Additional information on HRQOL calculations appears in eMethods 3 and eTables 3 to 5 in Supplement 1. We also conservatively assumed that during graft failure (and prior to graft function for those who experienced DGF), a patient’s HRQOL was 0. In our probabilistic sensitivity analysis, we used a method to preserve rank ordering for age-specific HRQOL weights and time-since-transplant HRQOL weights.^24^ The inventory detailing the costs and HRQOL weights that we included can be found in eMethods 4 and eTable 6 in Supplement 1.

### Interventions

To evaluate hypothetical policies, we simulated an increased transplantation rate with a shift in the distribution of kidney quality due to the use of lower-quality but still acceptable-quality donor kidneys. The increased transplantation rate will likely depend on how transplant centers respond to currently hypothetical policies promoting the use of additional acceptable-quality kidneys. Hence, we considered the status quo (0% increase in transplantation rate) along with policies that would increase the transplantation rate by 5%, 10%, 15%, 20%, or 25%. We assumed that with the implementation of these policies, the average quality of deceased donor kidneys would decline to closely resemble the distribution of kidney quality as measured by the kidney donor profile index (KDPI) used in France’s system. The KDPI represents the risk of graft loss, with higher values reflecting higher risk and associated with a higher probability of DGF.^25^ Thus, the KDPI distribution under the policies reflects greater use of high-KDPI donor kidneys. It is likely that a currently used kidney of a given KDPI is of higher quality than an unused kidney of the same KDPI because there are other characteristics not captured in the KDPI that can contribute to the quality of the donor kidney. We reflected this possibility by assuming that some proportion of newly transplanted kidneys would be lower quality by increasing their KDPI by 5 KDPI percentage points for 5% of donor kidneys. We conducted scenario analyses around the proportion of donor kidneys for which we increased the KDPI in 5% increments for up to 20% of donor kidneys.

### Statistical Analysis

Statistical analysis was performed from January 2023 to December 2025. Our primary outcomes include discounted lifetime costs, quality-adjusted life-years (QALYs), and incremental cost-effectiveness ratios (ICERs). We report rates of key waiting list and posttransplant outcomes. We performed analyses from the health care sector perspective and from the modified health care sector perspective (including patient time and caregiving costs). The analyses had a lifetime horizon with future costs and QALYs discounted at 3% annually.^26^ All costs are inflation adjusted to 2023 US dollars using the Personal Consumption Expenditure Health Total Deflator for all costs except wage estimates, for which we used the Consumer Price Index.^27,28^ We evaluated the cost-effectiveness of interventions using a $100 000 per QALY gained willingness-to-pay (WTP) threshold.^29^ We present results for the entire older adult transplant candidate population and for key subgroups (age, designated race and ethnicity [Hispanic, non-Hispanic Black, non-Hispanic White, and non-Hispanic other race or ethnicity (all non-Hispanic races and ethnicities that are not Black or White)], and diabetes status). Race and ethnicity were modeled because prior literature documents disparities in chronic kidney disease, dialysis, and transplant outcomes across different designated races and ethnicities of patients.^30,31,32,33,34,35,36,37^ Studies have also found that non-Hispanic Black patients have better expected survival with dialysis and worse expected posttransplant outcomes compared with non-Hispanic White patients.^38,39^

We explored uncertainty using probabilistic sensitivity analysis (PSA), visualizing the optimal strategy at various WTP thresholds using cost-effectiveness acceptability curves and the cost-effectiveness acceptability frontier. Expected loss curves showed the expected forgone benefits associated with choosing a suboptimal strategy depending on the WTP threshold.^40^ eTables 1, 3, and 4 and eMethods 5 in Supplement 1 provide additional details on the PSA. Analyses were performed using R programming language, version 4.4.0 (R Project for Statistical Computing).

## Results

### Base Case Results

The characteristics of the synthetic cohort of 100 000 individuals (mean age, 68.8 years [95% CI, 65.0-78.0 years]; 61.7% men and 38.3% women; 13.4% Hispanic, 26.1% non-Hispanic Black, 50.6% non-Hispanic White, and 9.9% non-Hispanic other race or ethnicity) are shown in eTable 7 in Supplement 1. The cohort had received dialysis for a mean of 1.2 years (95% CI, 0-6.6 years) and 56.8% had diabetes.

Increasing the deceased donor transplantation rate was associated with substantially decreased deaths on the waiting list and decreased waiting list removals as well as increased total net number of people undergoing transplants (Table 2). Using lower-quality donor kidneys was associated with an increased risk of DGF, but shorter wait times were associated with a decrease in this risk. At a 5% increase in the transplantation rate, the DGF rate increased from the status quo, likely due to small reductions in waiting times. However, as the transplantation rate increased to 25%, the DGF rate moved back toward the status quo because of offsetting risk factors. This pattern also occurred for the graft failure rate. Although lower kidney quality was associated with higher graft failure risk, shorter pretransplant dialysis time had a protective association.

**Table 2.** Base-Case Event Counts

Event	Status quo, event count (range)	Incremental difference in event counts by increase in rate of deceased donor transplant				
		5%	10%	15%	20%	25%
Rate per 10 000 kidney transplant candidates						
Deceased donor transplant	3623 (3265 to 3898)	123 (114 to 130)	241 (226 to 252)	356 (338 to 368)	468 (444 to 486)	576 (546 to 598)
Living donor transplant	1330 (1162 to 1542)	−13 (−16 to −8)	−25 (−29 to −20)	−37 (−42 to −31)	−49 (−55 to −42)	−60 (−69 to −52)
Waiting list deaths	1640 (1452 to 1799)	−30 (−35 to −26)	−59 (−67 to −50)	−87 (−100 to −72)	−114 (−129 to −98)	−141 (−161 to −118)
Other waiting list removals	3407 (3163 to 3643)	−80 (−89 to −74)	−157 (−167 to −144)	−232 (−246 to −216)	−305 (−321 to −283)	−375 (−396 to −350)
Delayed graft function (range), per 10 000 deceased donor transplants	2941 (729 to 5985)	26 (−26 to 85)	20 (−34 to 78)	13 (−40 to 65)	7 (−45 to 66)	1 (−50 to 59)
Graft loss (range), per 10 000 deceased donor transplants	1343 (231 to 2852)	47 (−4 to 163)	47 (−3 to 163)	47 (−2 to 168)	48 (−3 to 170)	49 (−4 to 173)

**Figure 2.**
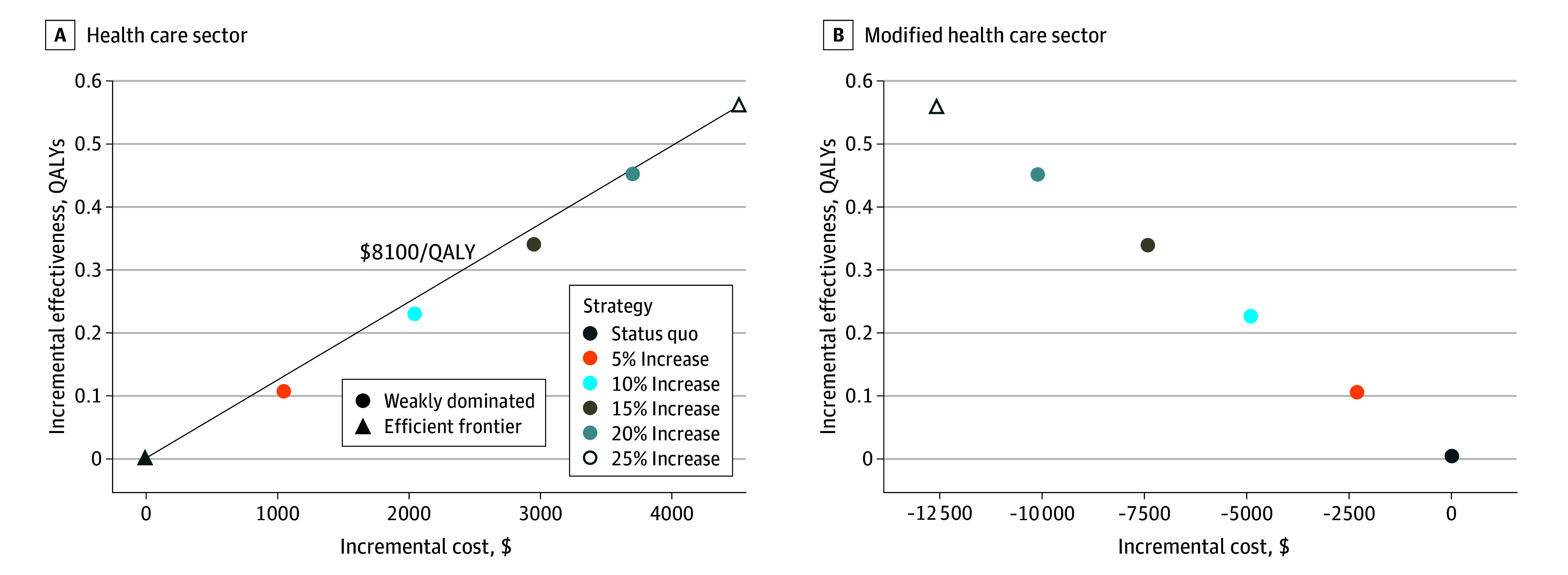
Incremental Cost-Effectiveness Frontiers The frontiers show the base-case results from the health care sector perspective (A) and the modified health care sector perspective (B). QALYs indicates quality-adjusted life-years.

Increasing the deceased donor transplantation rate by 25% for older transplant candidates using acceptable-quality kidneys was associated with an increased HRQOL cost-effectively. It would cost $8100 (95% credible interval [CrI], $700-$14 100) per QALY gained from the health care sector perspective (Figure 2). Increasing the transplantation rate by smaller amounts cost more per QALY gained than increasing by 25% because smaller increases not only mean fewer people undergo transplants but also the mean time to transplant is longer, resulting in additional adverse outcomes and costs.

From the modified health care sector perspective, increasing the deceased donor transplantation rate by 25% was associated with the most QALYs and lowest costs. Compared with the status quo, older candidates would be expected to gain 0.56 QALYs (95% CrI, 0.35-0.90 QALYs) and save $10 200 (95% CrI, $3400-$17 900). Additional results displayed using incremental net monetary benefit can be found in eFigures 4 to 8 in Supplement 1.

### Subgroups

Although older transplant candidates have shorter life expectancy and worse expected outcomes than younger candidates both while undergoing dialysis and after transplant, we found that even for individuals aged 75 years or older, increasing deceased donor transplants by 25% was associated with HRQOL gains. It also cost $7800 (95% CrI, $1300-$14 500) per QALY gained (eFigure 9 in Supplement 1).

Increasing deceased donor transplants by 25% was associated with substantial health benefits for patients of all designated races and ethnicities. Non-Hispanic Black patients have a longer-than-average duration on the waiting list; as such, increasing transplantation rates is associated with additional benefits. From the health care sector perspective, transplants for patients from all racial and ethnic subgroups cost far less than the $100 000 per QALY gained WTP threshold (eFigure 10 in Supplement 1). Gains associated with increasing transplantation rates are cost-effective for all groups from the health care sector perspective and cost saving from the modified health care sector perspective and would enhance health equity.

Transplant candidates with diabetes have a shorter life expectancy and worse health outcomes than candidates without diabetes. For individuals with diabetes, increasing the deceased donor transplantation rate by 25% was associated with HRQOL gains, albeit smaller than for those without diabetes. For those with diabetes, it would cost $9900 (95% CrI, $2500-$16 400) per QALY gained from the health care sector perspective (eFigure 11 in Supplement 1).

### Scenario Analysis

It is likely that some kidneys that are currently used for transplants are lower quality than other kidneys that are used, even if they have the same KDPI. Because we do not know exactly how many kidneys are worse and by how much, we varied our assumption in a scenario analysis. If as many as 20% of transplanted kidneys are of lower quality than their KDPI implies, increasing the transplantation rate by 25% would be associated with 141 (range, 118-161) averted waiting list deaths and 375 (range, 350-396) waiting list removals per 10 000 candidates and 175 (range, 25-385) additional DGF cases and 103 (range, 9-266) additional graft loss events per 10 000 recipients. In this scenario, increasing the transplantation rate by 25% remains cost-effective from the health care sector perspective (eFigure 12 in Supplement 1).

### Probabilistic Sensitivity Analysis

**Figure 3.**
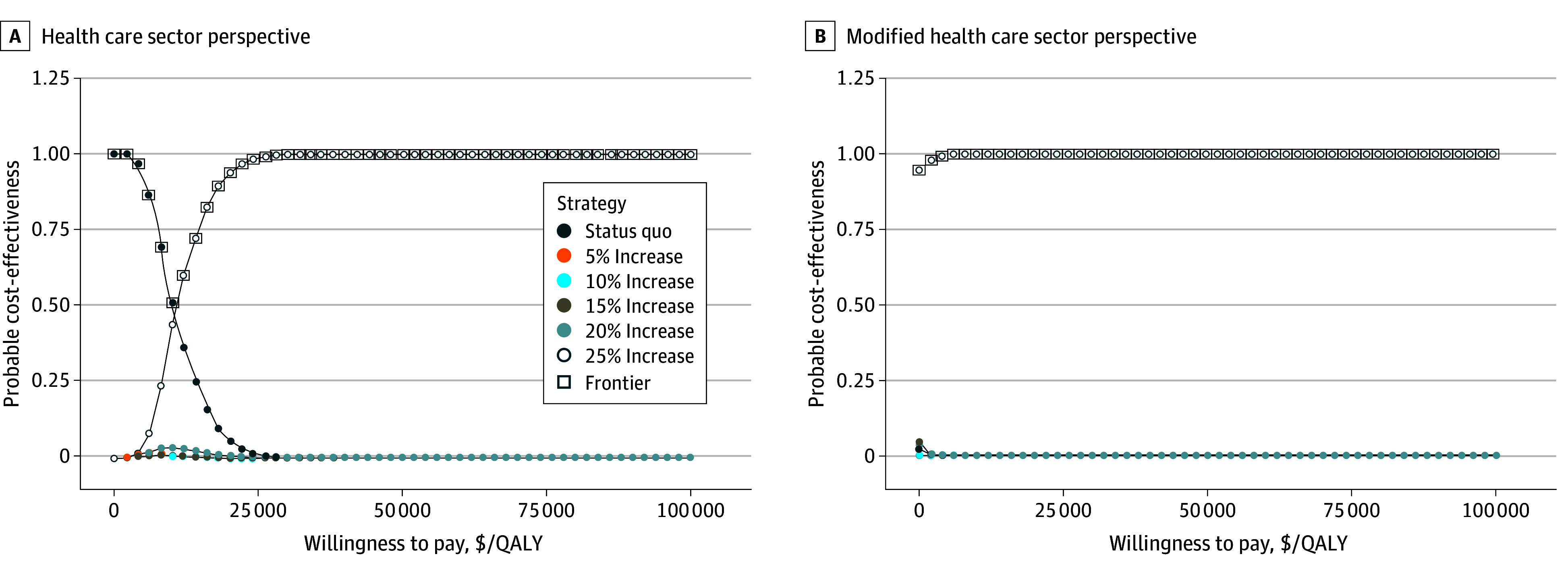
Cost-Effectiveness Acceptability Curves and Cost-Effectiveness Acceptability Frontier Results are from the health care sector perspective (A) and the modified health care sector perspective (B). QALY indicates quality-adjusted life-year.

At a WTP threshold of $100 000 per QALY gained, increasing the transplantation rate by 25% was cost-effective in 100% of the PSA samples from both the health care sector and modified health care sector perspectives (Figure 3). It is only for WTP thresholds below $40 000 per QALY gained that the status quo transplantation rate was preferred in any of the samples from the health care sector perspective. From the modified health care sector perspective, the 25% increase was cost saving in 77% of PSA samples at the $0 per QALY gained threshold. At a WTP threshold of $100 000 per QALY gained, choosing the status quo instead of increasing the transplantation rate by 25% would result in an expected loss of $51 400 per person, for a total of $886 million, from the health care sector perspective, and $58 300 per person, for a total of $1 billion, from the modified health care sector perspective (eFigure 13 in Supplement 1).

## Discussion

Increasing the deceased donor kidney transplantation rate for older candidates was associated with fewer adverse waiting list events and increased survival and HRQOL. We estimate that there would be fewer waiting list deaths and waiting list removals among older candidates, with modest increases in the rate of DGF and graft loss events. Although current kidney allocation decisions are not made based on cost, increasing the transplantation rate in this manner can be done in a cost-effective way. When we consider additional costs to society, such as caregiver and patient time, the policy was estimated to be cost saving. Using acceptable-quality deceased donor kidneys was associated with health benefits for the recipient and shorter wait times for other candidates.

Acceptable-quality kidneys can be thought of as kidneys across the KDPI distribution that could be used for transplants but, under the current allocation system, are not; use of acceptable-quality kidneys would also be dependent on the clinical context. Potential policies aimed at facilitating older candidates’ access to more acceptable-quality kidneys should be considered. Our analysis does not address the issues that patients with ESKD face related to financial and practical barriers in access to care and does not suggest that older candidates should be waitlisted for transplants. The current system is constrained in such a way that patient preferences are not always easy to fully incorporate into the decision-making process beyond whether to accept offers for high-KDPI kidneys. Although many older patients would prefer a kidney transplant if available, the decision might be informed by factors including how well they are faring on dialysis, underlying diseases that modify the risk of complications of immunosuppression, or other factors unique to each patient.^41^ Older transplant candidates have shown a willingness to accept lower kidney quality in order to have less wait time, hence the reason our analysis focuses on this population.^8^ However, we believe that access to any acceptable-quality kidneys should be extended to any candidate who expresses this preference. If patients are informed of the risks associated with receiving a lower-quality donor kidney, transplant centers should not be penalized for honoring their preferences. Recent meetings on stakeholder perspectives resulted in recommendations that include shared decision-making around whether to accept offers from medically complex donors.^42^ We believe that there is room for policies that allow older candidates access to such organs without being constrained by performance metrics. One such policy involves exempting older candidates willing to accept offers for lower-quality donor kidneys from transplant center performance metrics, reducing the incentives for transplant centers to optimize performance metrics. Given that performance metrics are currently under review, there is an opportunity to make it easier to honor patient preferences.

### Strengths and Limitations

Our study has some strengths, including the use of individual-level characteristics to parameterize the simulation model and build the synthetic cohort. By using individual-level data on all older transplant candidates in the US, the model can appropriately reflect the heterogeneous risks of outcomes for individuals. We also performed a full probabilistic analysis based on established calibration methods, allowing us to reflect the uncertainty in outcomes consistent with the empirical data.^10,43^ Even with substantial uncertainty and the inclusion of frequently omitted, substantial costs, our findings about the use of acceptable-quality kidneys are robust. Unlike earlier cost-effectiveness studies of different policies for kidney transplants, we included a more comprehensive assessment of costs, including those related to organ acquisition and opportunity costs experienced by patients and caregivers.

Our study also has some limitations, including that we did not consider downstream effects of the policies on younger candidates. If we had, we would expect to see younger candidates benefit from shorter waiting times for all candidates. Another limitation is that risks for simulated individuals in our model depend on race and ethnicity but do not depend on other socioeconomic measures. The underlying waiting list and transplant data do not have sufficient information on socioeconomic measures for us to include in the estimated risk equations. To the extent that designated race and ethnicity correlate with these socioeconomic factors, modeled differences in outcomes across race and ethnicity may capture some variability related to socioeconomic factors, as well as other social determinants of health, including systemic racism and other forms of discrimination.

## Conclusions

This economic analysis found that allowing older kidney transplant candidates access to acceptable-quality donor kidneys that currently go unused would improve health outcomes in a cost-effective manner. Doing so would also increase health equity, as older transplant candidates receive a disproportionally low number of transplants, despite stated preferences for accepting lower-quality kidneys to shorten these waits, leading to worse health outcomes. Our analysis suggests that decision-makers should consider policies that make better use of recovered kidneys to increase transplants among older patients and any other patients with similar preferences.

## Notes

Supplement 1. **eMethods 1.** Model Calibration **eTable 1.** Patient Characteristics at the Time of Transplant **eFigure 1.** Model Calibration Kaplan-Meier Curves for Waitlist Outcomes **eFigure 2.** Model Calibration Kaplan-Meier Curves for Posttransplant Outcomes **eFigure 3.** Model Calibration Plots for 30-Day Outcomes and Death at Graft Loss **eMethods 2.** Cost Parameters and Calculations **eTable 2.** Cost Inputs **eMethods 3.** Health-Related Quality of Life Calculations **eTable 3.** Longitudinal Health-Related Quality-of-Life Studies of Transplant Recipients **eTable 4.** Health-Related Quality of Life Weight Distribution Parameters **eTable 5.** Health-Related Quality of Life Inputs **eMethods 4.** Impact Inventory **eTable 6.** Impact Inventory **eMethods 5.** Analysis Methodology **eTable 7.** Baseline Characteristics **eFigure 4.** INMB by Perspective **eFigure 5.** INMB by Age Group **eFigure 6.** INMB by Diabetes Status **eFigure 7.** INMB by Race and Ethnicity Subgroup **eFigure 8.** INMB for Scenario Analysis on Kidney Quality **eFigure 9.** Incremental Cost-Effectiveness Frontier by Age Group and Perspective **eFigure 10.** Incremental Cost-Effectiveness Frontier by Race and Ethnicity Group and Perspective **eFigure 11.** Incremental Cost-Effectiveness Frontier by Diabetes History and Perspective **eFigure 12.** Incremental Cost-Effectiveness Frontier by Perspective and the Percentage of Kidneys With Worse Quality Than Their KDPI Implies **eFigure 13.** Expected Loss Curves by Perspective
eReferences.

Supplement 2.
Data Sharing Statement
