# Supplementary material for: Cost-Effectiveness of Acceptable-Quality Deceased Donor Kidneys for Transplant in Older Candidates
Source: JAMA Netw Open. 2026 Jan 27;9(1):e2555428. doi: 10.1001/jamanetworkopen.2025.55428 (PMC12848627; doi:10.1001/jamanetworkopen.2025.55428)
Supplement: Supplement 1. — eMethods 1. Model Calibration eTable 1. Patient Characteristics at the Time of Transplant eFigure 1. Model Calibration Kaplan-Meier Curves for Waitlist Outcomes eFigure 2. Model Calibration Kaplan-Meier Curves for Posttransplant Outcomes eFigure 3. Model Calibration Plots for 30-Day Outcomes and Death at Graft Loss eMethods 2. Cost Parameters and Calculations eTable 2. Cost Inputs eMethods 3. Health-Related Quality of Life Calculations eTable 3. Longitudinal Health-Related Quality-of-Life Studies of Transplant Recipients eTable 4. Health-Related Quality of Life Weight Distribution Parameters eTable 5. Health-Related Quality of Life Inputs eMethods 4. Impact Inventory eTable 6. Impact Inventory eMethods 5. Analysis Methodology eTable 7. Baseline Characteristics eFigure 4. INMB by Perspective eFigure 5. INMB by Age Group eFigure 6. INMB by Diabetes Status eFigure 7. INMB by Race and Ethnicity Subgroup eFigure 8. INMB for Scenario Analysis on Kidney Quality eFigure 9. Incremental Cost-Effectiveness Frontier by Age Group and Perspective eFigure 10. Incremental Cost-Effectiveness Frontier by Race and Ethnicity Group and Perspective eFigure 11. Incremental Cost-Effectiveness Frontier by Diabetes History and Perspective eFigure 12. Incremental Cost-Effectiveness Frontier by Perspective and the Percentage of Kidneys With Worse Quality Than Their KDPI Implies eFigure 13. Expected Loss Curves by Perspective eReferences. [file jamanetwopen-e2555428-s001.pdf]

Supplementary Online Content

Kaufmann MB, Tan JC, Owens DK, Chertow GM, Goldhaber-Fiebert JD. Cost-effectiveness of acceptable-quality deceased donor kidneys for transplant in older candidates. *JAMA Netw Open.* 2026;9(1):e2555428. doi:10.1001/jamanetworkopen.2025.55428

eMethods 1. Model Calibration
eTable 1. Patient Characteristics at the Time of Transplant
eFigure 1. Model Calibration Kaplan-Meier Curves for Waitlist Outcomes
eFigure 2. Model Calibration Kaplan-Meier Curves for Posttransplant Outcomes
eFigure 3. Model Calibration Plots for 30-Day Outcomes and Death at Graft Loss
eMethods 2. Cost Parameters and Calculations
eTable 2. Cost Inputs
eMethods 3. Health-Related Quality of Life Calculations
eTable 3. Longitudinal Health-Related Quality-of-Life Studies of Transplant Recipients
eTable 4. Health-Related Quality of Life Weight Distribution Parameters
eTable 5. Health-Related Quality of Life Inputs
eMethods 4. Impact Inventory
eTable 6. Impact Inventory
eMethods 5. Analysis Methodology
eTable 7. Baseline Characteristics
eFigure 4. INMB by Perspective
Figure 5. INMB by Age Group
eFigure 6. INMB by Diabetes Status
eFigure 7. INMB by Race and Ethnicity Subgroup
eFigure 8. INMB for Scenario Analysis on Kidney Quality
eFigure 9. Incremental Cost-Effectiveness Frontier by Age Group and Perspective
eFigure 10. Incremental Cost-Effectiveness Frontier by Race and Ethnicity Group and Perspective
eFigure 11. Incremental Cost-Effectiveness Frontier by Diabetes History and Perspective
eFigure 12. Incremental Cost-Effectiveness Frontier by Perspective and the Percentage of Kidneys With Worse Quality Than Their KDPI Implies
eFigure 13. Expected Loss Curves by Perspective
eReferences.

This supplementary material has been provided by the authors to give readers additional information about their work.

© 2026 Kaufmann MB et al. *JAMA Network Open.*

eMethods 1. Model Calibration

After the publication of the manuscript that describes our model development and calibration, we sought to improve the fit of the model with a focus on post-transplant outcomes.[10] We changed our goodness-of-fit metric from a sum of squared errors to a likelihood-based approach. The targets are derived from the data that was held out from equation development. For the survival equations, we tested two methods of calibration. The first was to use points along the Kaplan-Meier curves, assuming a normal distribution around the point estimates. The second method was to fit parametric regressions without covariates to the held-out data and use the auxiliary parameters as the targets. We then assumed a multivariate normal distribution around the auxiliary parameters. For the 30-day outcomes that are estimated using a multinomial logistic regression, our targets assumed a Dirichlet distribution. For death the same day as graft loss, which we estimate using a logistic regression, we assumed a binomial distribution. We also added additional calibration targets for the patient characteristics at the time of transplant. These characteristics include age at transplant, sex, race/ethnicity, blood type, years on dialysis before transplant, diabetes history, chronic obstructive pulmonary disease (COPD) history, peripheral vascular disease (PVD) history, angina/ coronary artery disease (CAD) history, peak calculated panel reactive antibodies (cPRA), and Organ Procurement and Transplantation Network (OPTN) region. We assumed normal distributions for each of these characteristics. We accounted for temporal changes in the time-to-event equations by including a coefficient for the year of listing in waitlist outcome equations.

Another change to our calibration method was to conduct the calibration in two stages: waitlist outcomes and post-transplant outcomes. Once we have a sufficient number of parameter sets that fit the waitlist outcomes well, we sample from those parameter sets of waitlist equations

© 2026 Kaufmann MB et al. *JAMA Network Open.*

and combine them with parameter sets for the post-transplant outcome equations. We do this because the accuracy of the post-transplant outcomes is dependent on first predicting the correct mix of patients who receive a deceased donor kidney. We generated 100,000 parameter sets and identified 64 parameter sets that we deemed to be acceptable.

eTable 1 shows patient characteristics at the time of transplant for the observed data compared to our calibrated parameter sets. **eFigures 1-3** show how the simulated outcomes compare to those in the held-out observed data. We can see that we have good fit to the observed data. Compared to the original calibrated parameter sets, we see the most improvement for our death after graft loss and 30-day outcomes equations.

© 2026 Kaufmann MB et al. *JAMA Network Open.*

eTable 1. Patient Characteristics at the Time of Transplant

	Observed	Simulated (95% CrI)
Age at Transplant	69.3	70.44 (70.33 - 70.56)
Sex	0.62	0.62 (0.61 - 0.62)
White	0.51	0.49 (0.48 - 0.5)
Black	0.25	0.28 (0.27 - 0.28)
Hispanic	0.14	0.13 (0.13 - 0.13)
Other Race/Ethnicity	0.1	0.1 (0.1 - 0.11)
Blood A	0.37	0.36 (0.35 - 0.36)
Blood AB	0.05	0.05 (0.05 - 0.05)
Blood B	0.14	0.14 (0.13 - 0.15)
Blood O	0.44	0.45 (0.44 - 0.46)
Years on Dialysis Before Transplant	3.97	3.25 (3.19 - 3.32)
History of Diabetes	0.51	0.51 (0.5 - 0.52)
History of COPD	0.01	0.01 (0.01 - 0.01)
History of PVD	0.12	0.12 (0.12 - 0.12)
History of Angina/CAD	0.55	0.59 (0.58 - 0.6)
cPRA	0.17	0.17 (0.17 - 0.17)
OPTN 1	0.04	0.04 (0.04 - 0.04)
OPTN 2	0.14	0.13 (0.12 - 0.15)
OPTN 3	0.14	0.14 (0.14 - 0.14)
OPTN 4	0.07	0.06 (0.06 - 0.07)
OPTN 5	0.17	0.16 (0.15 - 0.17)
OPTN 6	0.05	0.06 (0.06 - 0.06)
OPTN 7	0.07	0.06 (0.05 - 0.06)
OPTN 8	0.07	0.08 (0.08 - 0.09)
OPTN 9	0.07	0.07 (0.06 - 0.07)
OPTN 10	0.08	0.08 (0.08 - 0.09)
OPTN 11	0.1	0.12 (0.11 - 0.12)

We compared the observed versus simulated patient characteristics and the 95% credible interval.

© 2026 Kaufmann MB et al. *JAMA Network Open.*

eFigure 1. Model Calibration Kaplan-Meier Curves for Waitlist Outcomes

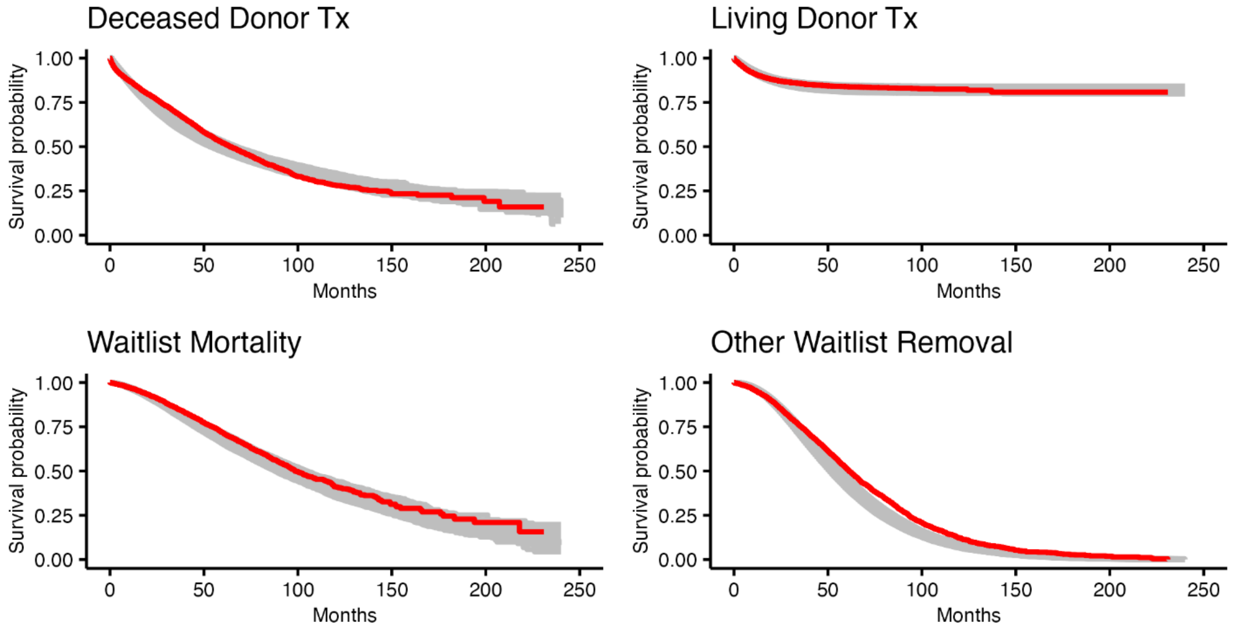

The Kaplan-Meier curves show simulated versus observed survival. The gray curves each represent 1 of the simulated best-fitting parameter sets, and the red curve represents the observed data.

© 2026 Kaufmann MB et al. *JAMA Network Open.*

eFigure 2. Model Calibration Kaplan-Meier Curves for Posttransplant Outcomes

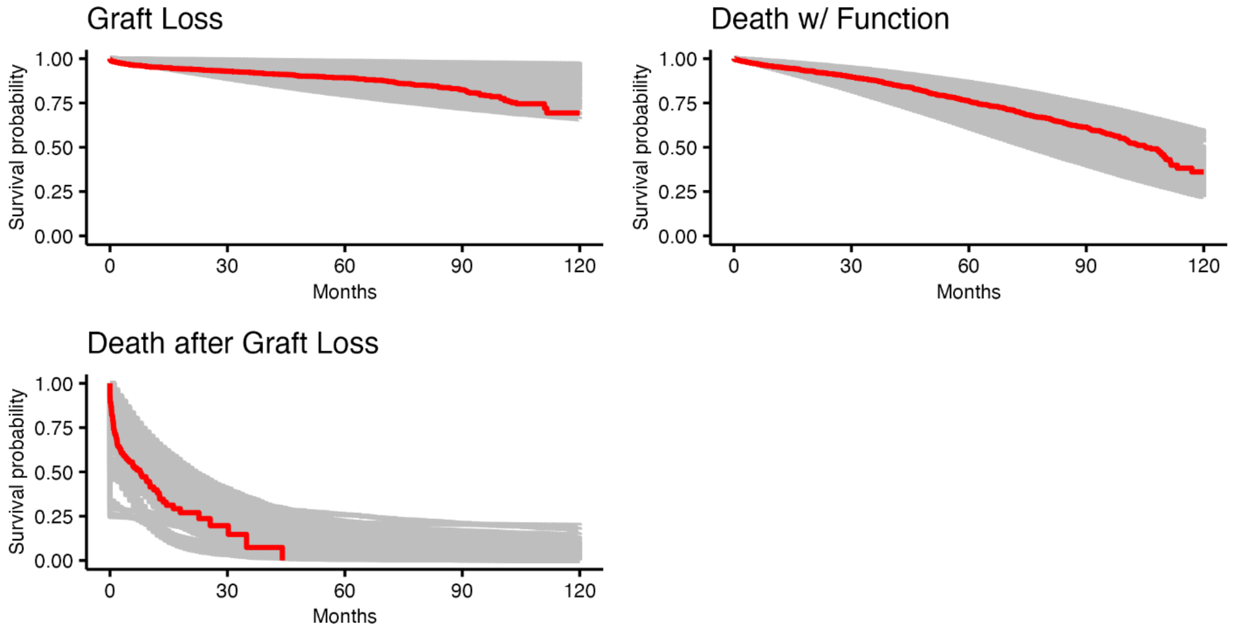

The Kaplan-Meier curves show simulated versus observed survival. The gray curves each represent 1 of the simulated best-fitting parameter sets, and the red curve represents the observed data.

© 2026 Kaufmann MB et al. *JAMA Network Open.*

eFigure 3. Model Calibration Plots for 30-Day Outcomes and Death at Graft Loss

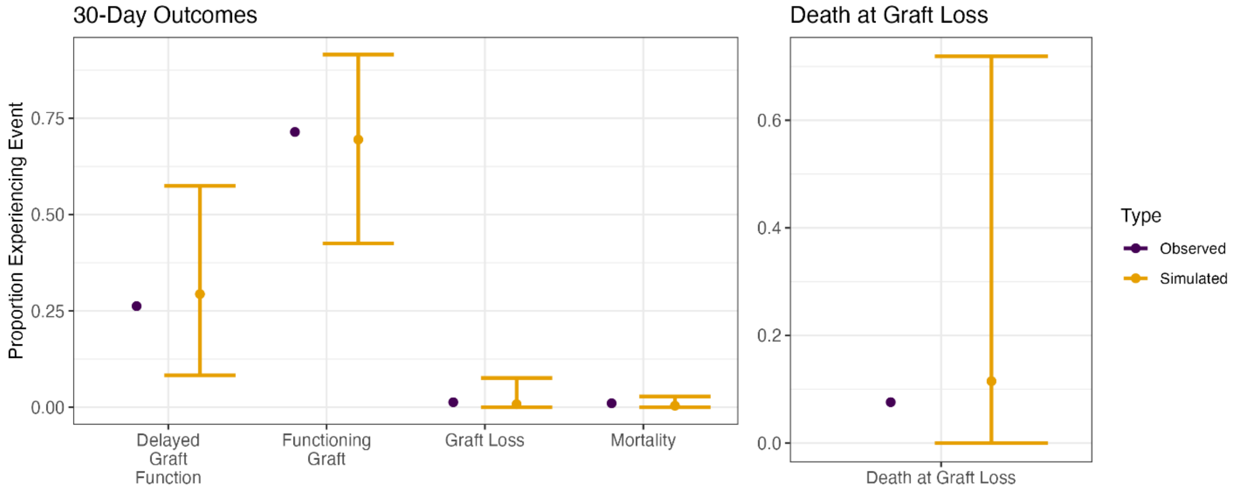

The 30-day outcomes were predicted using a multinomial logistic regression while death at graft loss was predicted using logistic regression. The purple represents the calibration targets while yellow represents the simulated mean and 95% credible intervals from the calibrated parameter sets.

eMethods 2. Cost Parameters and Calculations

The United States Renal Data System (USRDS) Annual Report provides total costs for dialysis, transplantation, and post-transplant costs which also include complications (including DGF and graft failure) that the model explicitly accounts for. These are based on Medicare costs, including Part D.[1] To avoid double counting the cost of complications, we disaggregated the total cost of transplantation into the initial hospitalization costs without complications and the cost of DGF. The estimated difference between hospital costs for recipients with and without DGF from Almond et al., 1991, is $15,760.[2] This value is 1989 USD, which we adjust to 2022 USD using the PCE-Health total, is $41,592.[3] Then we estimate the cost of deceased donor transplantation without delayed graft function (DGF). We start with our estimate cost of deceased donor transplantation from Axelrod et al., 2017.[4] The estimate provided, $106,675, is in 2013 USD. We then adjust this estimate to 2022 USD using the PCE-Health total over that period, which comes out to $125,127.[3] Next, we multiply the fraction of deceased donor kidney transplant recipients who experienced DGF by our DGF cost estimate.[2] Our final cost estimate for deceased donor transplantation is $125,127 – (0.251 * $41,592) = $114,688. We derived the cost of graft failure from the post-transplantation costs for a year in which graft failure occurs, by assuming that, on average, in the year of graft failure, the recipient has a functioning graft for 6 months, has the acute graft loss event during the next month, and 5 subsequent months of dialysis.[5]

For our probabilistic sensitivity analysis, the costs for each parameter set were drawn from the distributions described in **eTable 2**. For costs that are drawn from gamma distributions, we use Nelder-Mead optimization to calibrate the rate and shape parameters. We minimize the sum of squared errors of the mean and standard deviation of samples from a gamma distribution

© 2026 Kaufmann MB et al. *JAMA Network Open.*

and our mean and standard deviation targets. For costs that are drawn from a uniform distribution, we use +/- 20% of the point estimates.

Many of the cost inputs did not report uncertain ranges, standard deviations, or standard errors. Our approach to determining the standard deviations that we use as targets in the optimization algorithm is dependent on the source. For dialysis and post-transplant costs, we used the standard deviation of all the age-specific costs as the standard deviation for each individual age-specific cost. We use the same approach for the one-time graft loss event costs.

For our transplant costs we used the HCUPnet database as a means of determining the variation.[6] The standard error of DRG code 652, Kidney Transplant" was 3.03% of the point estimate. We assume that the standard deviation of both deceased donor and living donor transplants to be the same percentage of our point estimates. Because our DGF costs are a difference in means, we use the formula for the standard deviations of the difference of sample means:

$$\sigma_d = \sqrt{\sigma_1^2/n_1 + \sigma_2^2/n_2}$$

For caregiver time for patients on dialysis we fix the wage estimates and use the standard deviation of the caregiving hours. We multiply the standard deviation of the hours per month of caregiving time by the BLS estimate. For caregiver and patient time costs where literature estimates were not available, we had informal interviews with transplant nephrologists to estimate how many clinic visits and labs were required in each phase of the transplant process.

eTable 2 shows the cost parameters along with the distributions that are sampled from for the probabilistic sensitivity analysis.

© 2026 Kaufmann MB et al. *JAMA Network Open.*

eTable 2. Cost Inputs

Cost	Value	Distribution	Source(s)
**Healthcare Sector Perspective**			
Dialysis w/ Part D 65-69 (monthly)	$8,791	Gamma(shape = 3312.222, rate = 0.377)	2021 USRDS Annual Data Report
Dialysis w/ Part D 70-74 (monthly)	$8,675	Gamma(shape = 3206.55, rate = 0.37)	2021 USRDS Annual Data Report
Dialysis w/ Part D 75-79 (monthly)	$8,553	Gamma(shape = 3999.94, rate = 0.468)	2021 USRDS Annual Data Report
Dialysis w/ Part D 80-84 (monthly)	$8,672	Gamma(shape = 3211.89, rate = 0.37)	2021 USRDS Annual Data Report
Dialysis w/ Part D 85+ (monthly)	$8,387	Gamma(shape = 2993.366, rate = 0.357)	2021 USRDS Annual Data Report
Post-Transplant w/ Part D 65-69 (monthly)	$3,520	Gamma(shape = 476.943, rate = 0.135)	2021 USRDS Annual Data Report
Post-Transplant w/ Part D 70-74 (monthly)	$3,498	Gamma(shape = 470.804, rate = 0.135)	2021 USRDS Annual Data Report
Post-Transplant w/ Part D 75-79 (monthly)	$3,604	Gamma(shape = 1206.225, rate = 0.335)	2021 USRDS Annual Data Report
Post-Transplant w/ Part D 80-84 (monthly)	$3,419	Gamma(shape = 448.123, rate = 0.131)	2021 USRDS Annual Data Report
Post-Transplant w/ Part D 85+ (monthly)	$3,182	Gamma(shape = 1509.844, rate = 0.474)	2021 USRDS Annual Data Report
Deceased Donor Transplantation (one-time)	$114,687	Gamma(shape = 57402.46, rate = 0.5005)	Axelrod et al., 2017
Organ Acquisition Cost Center (one-time)	$112,318	Gamma(shape = 11.232, rate = 0.0001)	Cheng et al., 2022
Delayed Graft Function (one-time)	$41,592	Gamma(shape = 24.925, rate = 0.0006)	Almond et al., 1991
Graft Failure (one-time)	$85,989	Gamma(shape = 8.599, rate = 0.0001)	2021 USRDS Annual Data Report
Living Donor Transplant (one-time)	$111,976	Gamma(shape = 56062.28, rate = 0.5007)	Axelrod et al., 2017
Post-Waitlist Removal (multiplier)	1.2	Uniform(1.0, 1.4)	Assumed
**Societal Perspective**			
Caregiver Time			
Dialysis (monthly)	$5,867	Gamma(shape = 2934.9, rate = 0.05)	Liu et al., 2022, BLS May 2021
Post-Transplant (monthly)	$1,141	Gamma(shape = 2.0905, rate = 0.0018)	Langa et al., 2004, BLS May 2021
Patient Time			
Dialysis (monthly)	$1,611	Uniform(1289, 1933)	BLS May 2021, Expert Opinion
Post-Transplant: Month 1 (monthly)	$372	Uniform(298, 446)	BLS May 2021, Expert Opinion
Post-Transplant: Month 2-3 (monthly)	$186	Uniform(149, 223)	BLS May 2021, Expert Opinion
Post-Transplant: Month 4+ (monthly)	$62	Uniform(50, 74)	BLS May 2021, Expert Opinion

Note: Post-Transplant Removal multiplier is applied to dialysis costs to reflect that those removed from the waitlist are likely to have increased healthcare needs.

© 2026 Kaufmann MB et al. *JAMA Network Open.*

eMethods 3. Health-Related Quality of Life Calculations

The health-related quality of life weight calculations we are derived using two sources, Hanmer et al., 2006 and Wyld et al., 2012.[7,8] Wyld et al., 2012 is a meta-analysis of studies that estimate quality of life weights for kidney transplant candidates and recipients. From this paper, we use Table 4, which presents the quality-of-life weights from longitudinal studies that estimate the weights pre- and post-transplant (**eTable 3**).

© 2026 Kaufmann MB et al. *JAMA Network Open.*

eTable 3. Longitudinal Health-Related Quality-of-Life Studies of Transplant Recipients

Study	Utility Elicitation Instrument	Number of Patients	Utility					Mean Age
			Pre-Transplant	Post-Transplant				
				0—3 mo	4—8 mo	9-12 mo	13-24 mo	
Balaska et al. (20]	SF-36	85	0.35			0.6		43.8
Laupacis et al. (21]	TTO	131	0.57	0.71	0.75	0.74	0.7	42
Oberbauer et al. (22), group 1	SF-36	183		0.61		0.62	0.62	43.9
Oberbauer et al. (22), group 2	SF-36	178		0.61		0.6	0.6	45.2
Painter et al. (23), group 1	SF-36	14		0.59		0.58		48.3
Painter et al. (23), group 2	SF-36	9		0.67		0.69		46.8
Perez San Gregorio et al. (24)	SF-36	28	0.59	0.57	0.63	0.64		40.61
Pinson et al. (25)	SF-36	24	0.58	0.56				44
Ravagnani et al. [26)	SF-36	17	0.57				0.61	37.9
Rodrigue et al. (27)	SF-36	31	0.56	0.57	0.62	0.65		
Russell et al. [28]	TTO	27	0.41			0.74		41.9
Weighted Average		727	0.50	0.63	0.71	0.64	0.63	43.64

Adapted from Wyld et al., 2012 Table 4.

We derived these weights from studies with an average age younger than our population of interest. Because younger populations on average have higher health-related quality of life, we scaled these weights downward for our older population given that quality of life is lower in older ages.[9] We first calculated the difference between the quality of life weights of the population of transplant candidates and recipients and the general population of the same age. We then applied those differences to the age- and sex-specific quality of life weights that aligned with our population of candidates 65 and older.[9] To estimate age-, sex-, and time-since-transplant-specific weights, we used the Hanmer et al., 2006 EQ-5D estimates.[7] The studies for transplant related weights have an average age of about 44. We take the difference between these estimates and those of the general US population from ages 40-49 for males and females. Using the time-since-transplant differences from the general population, we subtract that difference

© 2026 Kaufmann MB et al. *JAMA Network Open.*

from the age ranges relevant to our study population (60-69, 70-79, and 80-89). These are our age-, sex-, and time-since-transplant-specific weights. We assume that those 90 and older have the same weights as those 80-89.

For our probabilistic sensitivity analysis, we draw from a beta distribution for our health-related quality of life weights. We estimate the beta distribution parameters using the estimated weights and the total sample size of the studies. They are calculated as:

$$\alpha = nw$$

$$\beta = n(1 - w)$$

where n is the sample size and w is the quality-of-life weight. Using the sample size of the studies in **eTable 3** ($n = 727$), the α and β parameters are shown in **eTable 4.**

© 2026 Kaufmann MB et al. *JAMA Network Open.*

eTable 4. Health-Related Quality of Life Weight Distribution Parameters

	Male					Female				
Age	Pre-Tx	0—3 mo	4—8 mo	9-12 mo	13-24 mo	Pre-Tx	0—3 mo	4—8 mo	9-12 mo	13-24 mo
**Alpha**										
60-69	334	422	480	436	429	327	414	480	429	422
70-79	305	393	458	407	400	298	385	451	400	393
80+	291	378	443	393	385	269	356	414	364	356
**Beta**										
60-69	395	306	244	294	301	398	309	248	298	304
70-79	422	333	272	322	328	427	338	277	327	334
80+	437	348	286	336	343	461	373	311	361	368

© 2026 Kaufmann MB et al. *JAMA Network Open.*

eTable 5. Health-Related Quality of Life Inputs

QALY	Age				Distribution	Source(s)
	All	65-69	70-79	80+		
Dialysis						
Male		0.46	0.42	0.40	Beta(alpha = (334, 305, 291) beta = (393, 422, 436))	Hanmer et al., 2006, Wyld et al., 2012
Female		0.43	0.39	0.34	Beta(alpha = (313, 284, 247) beta = 414, 443, 480))	Hanmer et al., 2006, Wyld et al., 2012
Post-Transplant (0-3 Months)						
Male		0.58	0.54	0.52	Beta(alpha = (422, 393, 378) beta = (305, 334, 349))	Hanmer et al., 2006, Wyld et al., 2012
Female		0.55	0.51	0.46	Beta(alpha = (400, 371, 334) beta = (327, 356, 393))	Hanmer et al., 2006, Wyld et al., 2012
Post-Transplant (4-8 Months)						
Male		0.66	0.63	0.61	Beta(alpha = (480, 458, 443) beta = (247, 269, 284))	Hanmer et al., 2006, Wyld et al., 2012
Female		0.64	0.60	0.55	Beta(alpha = (465, 436, 400) beta = (262, 291, 327))	Hanmer et al., 2006, Wyld et al., 2012
Post-Transplant (9-12 Months)						
Male		0.60	0.56	0.54	Beta(alpha = (436, 407, 393) beta = (291, 320, 334))	Hanmer et al., 2006, Wyld et al., 2012
Female		0.57	0.53	0.48	Beta(alpha = (414, 385, 393) beta = (313, 342, 378))	Hanmer et al., 2006, Wyld et al., 2012
Post-Transplant (13+ Months)						
Male		0.59	0.55	0.53	Beta(alpha = (429, 400, 385) beta = (298, 327, 342))	Hanmer et al., 2006, Wyld et al., 2012
Female		0.56	0.52	0.47	Beta(alpha = (407, 378, 342) beta = (320, 349, 385))	Hanmer et al., 2006, Wyld et al., 2012
Delayed Graft Function (one-time)	0					Assumed
Graft Failure (one-time)	0					Assumed

Note: we assume that patients who experience delayed graft function and graft failure derive 0 QALYs for the month in which these events occur.

© 2026 Kaufmann MB et al. *JAMA Network Open.*

eMethods 4. Impact Inventory

eTable 6. Impact Inventory

<table>
<tr><th rowspan="2">Sector</th><th rowspan="2">Type of Impact</th><th colspan="2">Included in this Reference Case Analysis From…Perspective?</th><th rowspan="2">Notes on Sources of Evidence</th></tr>
<tr><th>Health Care Sector</th><th>Societal</th></tr>
<tr><td colspan="5">Formal Health Care Sector</td></tr>
<tr><td rowspan="11">Health</td><td colspan="4">Health Outcomes (effects)</td></tr>
<tr><td>Longevity effects</td><td>Y</td><td>Y</td><td>Model equations account for life expectancy. Life expectancy after exiting model comes from 2021 USRDS annual data report</td></tr>
<tr><td>Health-related QoL effects</td><td>Y</td><td>Y</td><td>QALY estimates from Wyld et al. 2012 for transplant vs dialysis. QALY estimates are adjusted for age and sex</td></tr>
<tr><td>Other health effects (eg, adverse events)</td><td>Y</td><td>Y</td><td>Model equations track number of events for: transplants, waitlist and post-transplant mortality, waitlist removals, delayed graft function, and graft loss</td></tr>
<tr><td colspan="4">Medical Costs</td></tr>
<tr><td>Paid for by third-party payers</td><td>Y</td><td>Y</td><td>Medicare spending by modality and event type from 2021 USRDS annual data report</td></tr>
<tr><td>Paid for by patients out-of-pocket</td><td>N</td><td>N</td><td>Only using Medicare spending as for cost estimates</td></tr>
<tr><td>Future related medical costs (payers and patients)</td><td>Y</td><td>Y</td><td>2021 USRDS Annual Data Report has related medical costs</td></tr>
<tr><td>Future unrelated medical costs (payers and patients)</td><td>Y</td><td>Y</td><td>2021 USRDS Annual Data Report has related medical costs</td></tr>
<tr><td colspan="5">Informal Health Care Sector</td></tr>
</table>

© 2026 Kaufmann MB et al. *JAMA Network Open.*

Sector	Type of Impact	Included in this Reference Case Analysis From…Perspective?		Notes on Sources of Evidence
		Health Care Sector	Societal	
Health	Patient-time costs	NA	Y	Dialysis 3 Times a week, 4 hours each<br>Post-transplant time based on schedule of office visits and lab draws.
	Unpaid caregiver-time costs	NA	Y	Substantial caregiver-time pre-transplant, lower costs post-transplant
	Transportation costs	NA	N	Included in caregiver-time costs
Non-Health Care Sectors				
Productivity	Labor market earnings lost	NA	N	Older population assumed to be of retirement age
	Cost of unpaid lost productivity due to illness	NA	N	Older population assumed to be of retirement age
	Cost of uncompensated household production	NA	N	Older population assumed to be of retirement age
Consumption	Future consumption unrelated to health	NA	N	Not considered in our study
Social Services	Cost of social services as a part of intervention	NA	N	Not applicable
Legal or Criminal Justice	Number of crimes related to intervention	NA	N	Not applicable
	Cost of crimes related to intervention	NA	N	Not applicable
Education	Impact of intervention on educational achievement of population	NA	N	Not applicable to the older population
Housing	Cost of intervention on home improvements	NA	N	Assume to be in-center dialysis
Environment	Production of toxic waste pollution by intervention	NA	N	Not applicable
Other (specify)	Other impacts	NA	N	No other impacts identified

© 2026 Kaufmann MB et al. *JAMA Network Open.*

eMethods 5. Analysis Methodology

In our base case and scenario analyses, we simulated a population of 10,000,000 individuals per natural history parameter set from calibration to minimize stochastic noise due to simulating rare events. We calculated the difference in cost and quality of life weights for each parameter set, and then averaged over all the sets to determine cost-effectiveness and ICERs.[11]

We explored model uncertainty by running a probabilistic sensitivity analysis (PSA). For each of the 64 parameter sets from calibration, we combined them with 100 samples from the uncertainty distributions of our cost and QALY parameters. Further details on the distributions used for each input can be found in the Supplement, S1-S2. We than ran each of the 6,400 combinations of the model transition parameters, costs, and QALYs are then used to simulate 1,000,000 individuals to reduce the effect of first-order stochastic noise.[12]

Appendix Results

eTable 7 shows the baseline characteristics of the 100,000 transplant candidates that make up the synthetic cohort.

© 2026 Kaufmann MB et al. *JAMA Network Open.*

eTable 7. Baseline Characteristics

Candidate Baseline Characteristics	Candidates (N = 100,000)
Age at listing	68.8 (65.0 - 78.0)
Sex	
Male	61.7%
Female	38.3%
Race/Ethnicity	
Hispanic	13.4%
NH Black	26.1%
NH Other	9.9%
NH White	50.6%
Baseline Years on Dialysis	1.2 (0 - 6.6)
Calculated Panel Reactive Antibody (cPRA)	0.16 (0 - 0.98)
Blood Type	
A	32.4%
AB	3.5%
B	15.1%
O	49.0%
Comorbidities	
Diabetes	56.8%
Chronic Obstructive Pulmonary Disease (COPD)	1.5%
Peripheral Vascular Disease (PVD)	10.6%
Angina/Coronary Artery Disease (CAD)	59.3%

Baseline characteristics. For continuous variables we report the mean (2.5th and 97.5th percentiles) and for categorical/binary variables we report the proportion of the synthetic cohort in that category.

In our base-case results, we find that increasing the rate of deceased donor transplantation by 25% has an ICER of $8,100 per QALY gained compared to the status quo rate of transplantation. Smaller increases to the rate of transplantation are weakly dominated, but if we are unable to increase the rate of transplantation by 25%, it is important to quantify the impact of these smaller increases. From a societal perspective, any increases in the rate of transplantation are considered cost-saving. If we present cost-saving interventions as ICERs, they have negative values, which are indiscernible from interventions that are more costly and less effective. To

© 2026 Kaufmann MB et al. *JAMA Network Open.*

overcome this, we can use a commonly used metric in cost-effectiveness research, incremental net monetary benefit (INMB).

$$INMB = \lambda \times \Delta\bar{E} - \Delta\bar{C}$$

where λ is the willingness-to-pay (WTP) thershold, $\Delta\bar{E}$ is incremental difference in mean health effects, and $\Delta\bar{C}$ is the difference in mean costs. We use analysis of variance (ANOVA) to test for differences in INMB between scenarios and the Wilcoxon test when comparing INMB between two subgroups.

Using INMB, increasing the rate of transplantation to 25% results in the highest expected benefit, but all rates results positive INMB. This means that we should increase the rate of transplantation as much possible using imperfect but transplant quality kidneys. The INMB for increasing the rate of transplantation by 25% is $52,800 (95% CrI: $35,500-$80,800) and $66,100 (95% CrI: $47,700-$91,800) from the healthcare sector and societal perspectives, respectively (**eFigure 4**). The expected INMB of increasing the rate of transplantation by 25% is somewhat smaller with older ages but substantially greater than 0 and hence remains the preferred strategy (**eFigure 5**). Candidates with diabetes have lower, but still positive, expected INMB from both perspectives (**eFigure 6**). We also find that compared to NH White candidates, all other race/ethnicity groups benefit more from a higher rate of transplantation (**eFigure 7**).

© 2026 Kaufmann MB et al. *JAMA Network Open.*

eFigure 4. INMB by Perspective

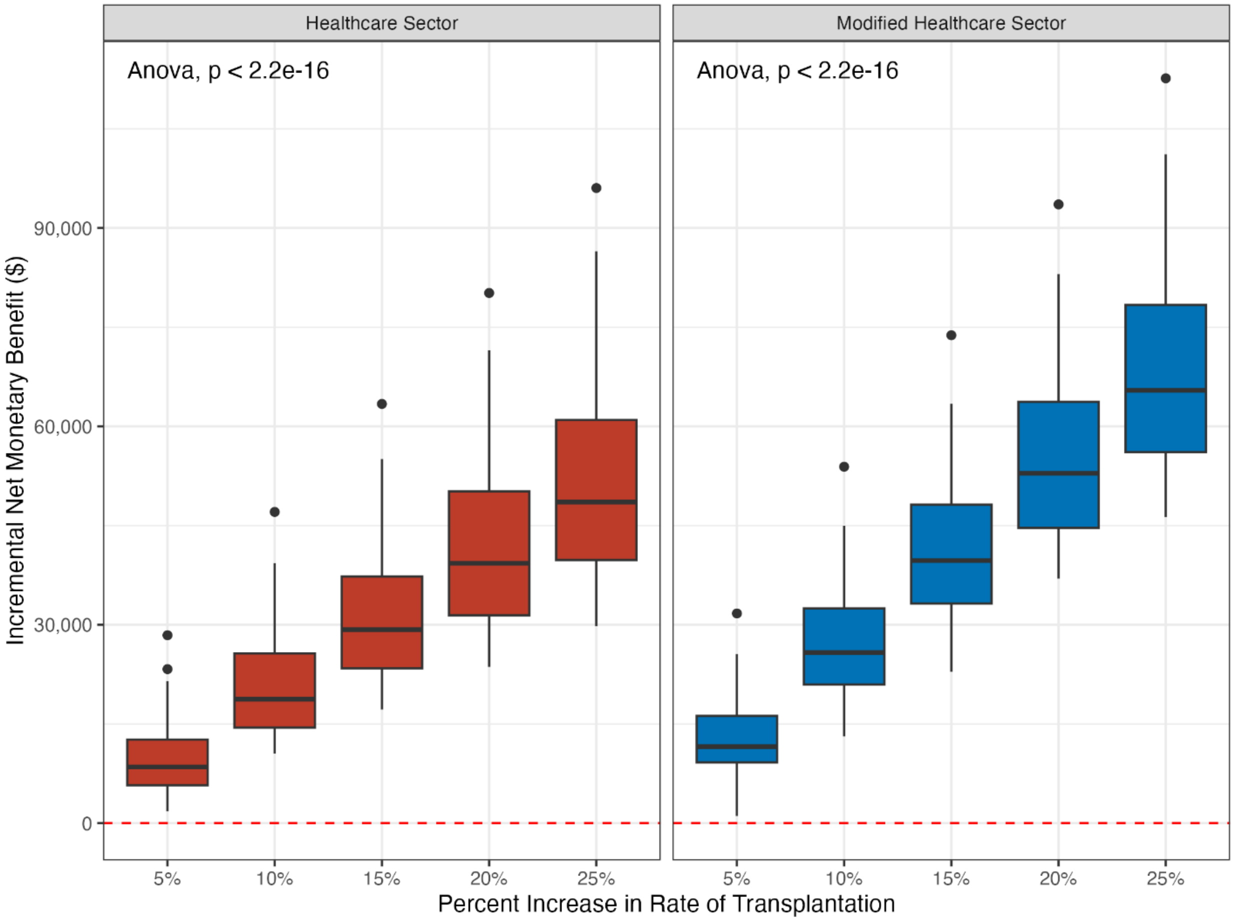

A comparison of means is tested using ANOVA tests, where the null hypothesis is that there is no difference in the mean INMB across the increasing rates of transplantation.
INMB: Incremental net monetary benefit

© 2026 Kaufmann MB et al. *JAMA Network Open.*

eFigure 5. INMB by Age Group

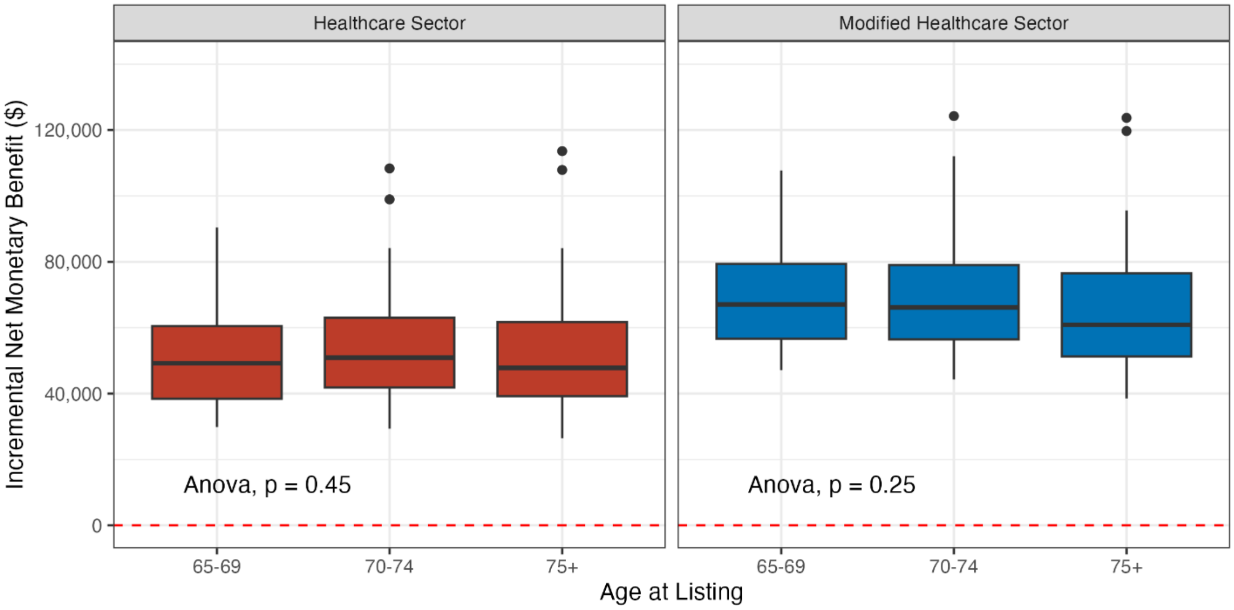

A comparison of means is tested using a ANOVA test, where the null hypothesis is that there is no difference in the mean INMB between the two subgroups. There is no statistically significant difference in INMB by age group.
INMB: Incremental net monetary benefit

© 2026 Kaufmann MB et al. *JAMA Network Open.*

eFigure 6. INMB by Diabetes Status

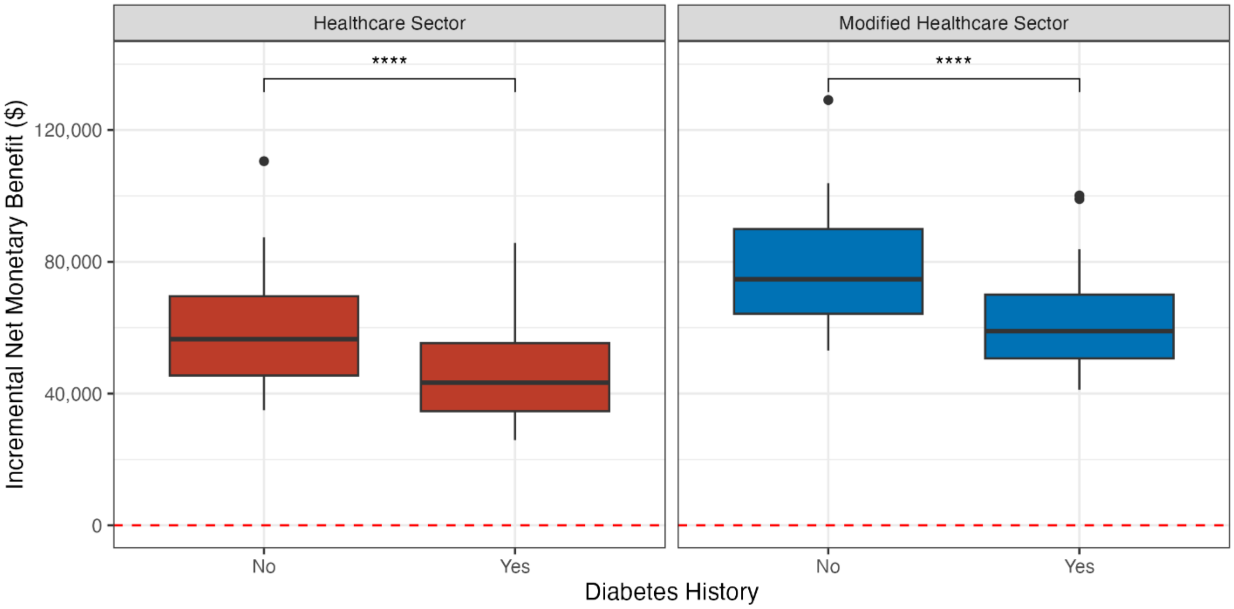

A comparison of means is tested using a Wilcoxon test, where the null hypothesis is that there is no difference in the mean INMB between the two subgroups. Statistical significance symbols: **** p ≤ 0.0001.
INMB: Incremental net monetary benefit

© 2026 Kaufmann MB et al. *JAMA Network Open.*

eFigure 7. INMB by Race and Ethnicity Subgroup

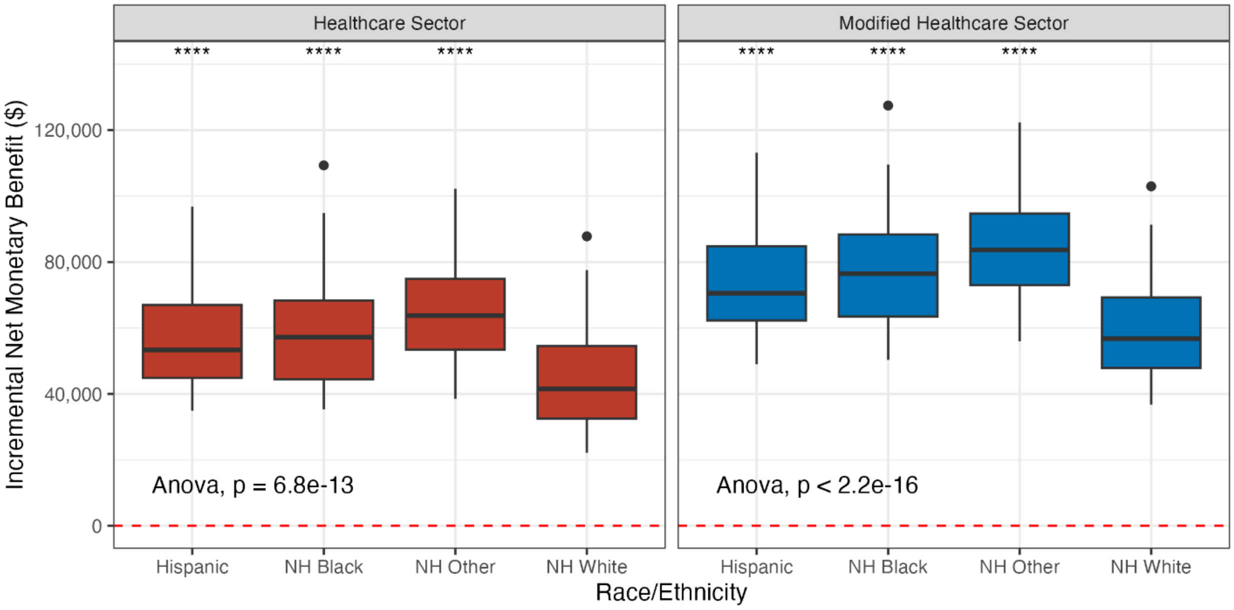

A comparison of means is tested using a Wilcoxon test, where the null hypothesis is that there is no difference in the mean INMB between NH White candidates and each other race/ethnicity group. Statistical significance symbols: **** p ≤ 0.0001.
INMB: Incremental net monetary benefit

© 2026 Kaufmann MB et al. *JAMA Network Open.*

eFigure 8. INMB for Scenario Analysis on Kidney Quality

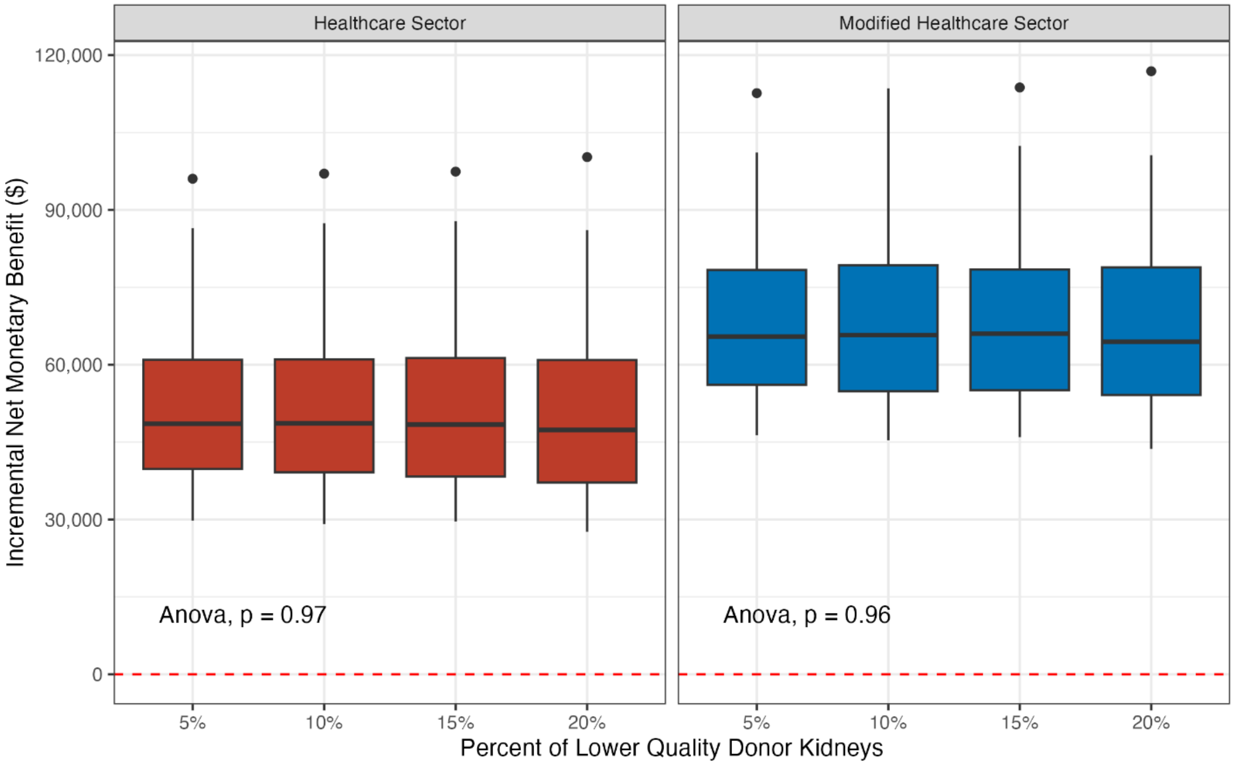

A comparison of means using ANOVA tests, where the null hypothesis is that there is no difference in the mean INMB across the percentage of kidneys with lower quality.
INMB: Incremental net monetary benefit

© 2026 Kaufmann MB et al. *JAMA Network Open.*

eFigure 9. Incremental Cost-Effectiveness Frontier by Age Group and Perspective

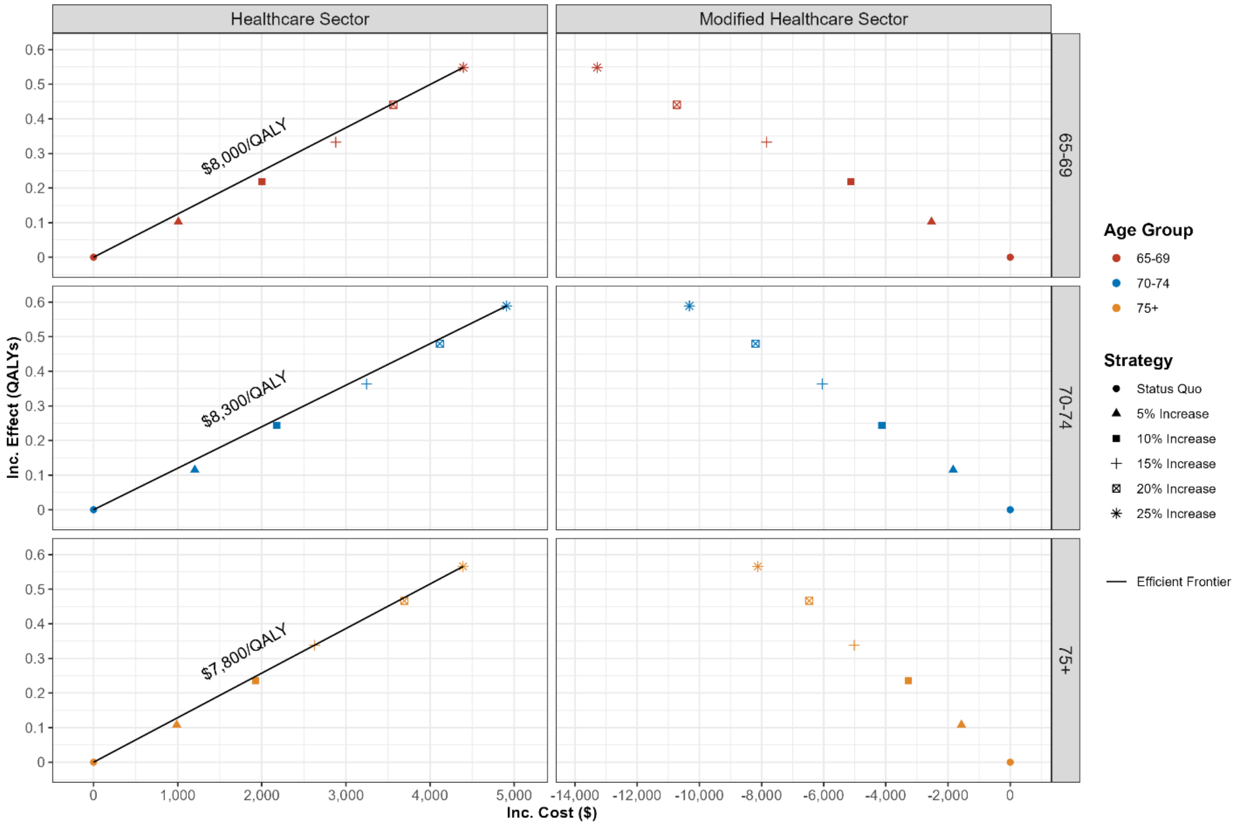

QALYs: quality-adjusted life years
Inc: Incremental

© 2026 Kaufmann MB et al. *JAMA Network Open.*

eFigure 10. Incremental Cost-Effectiveness Frontier by Race and Ethnicity Group and Perspective

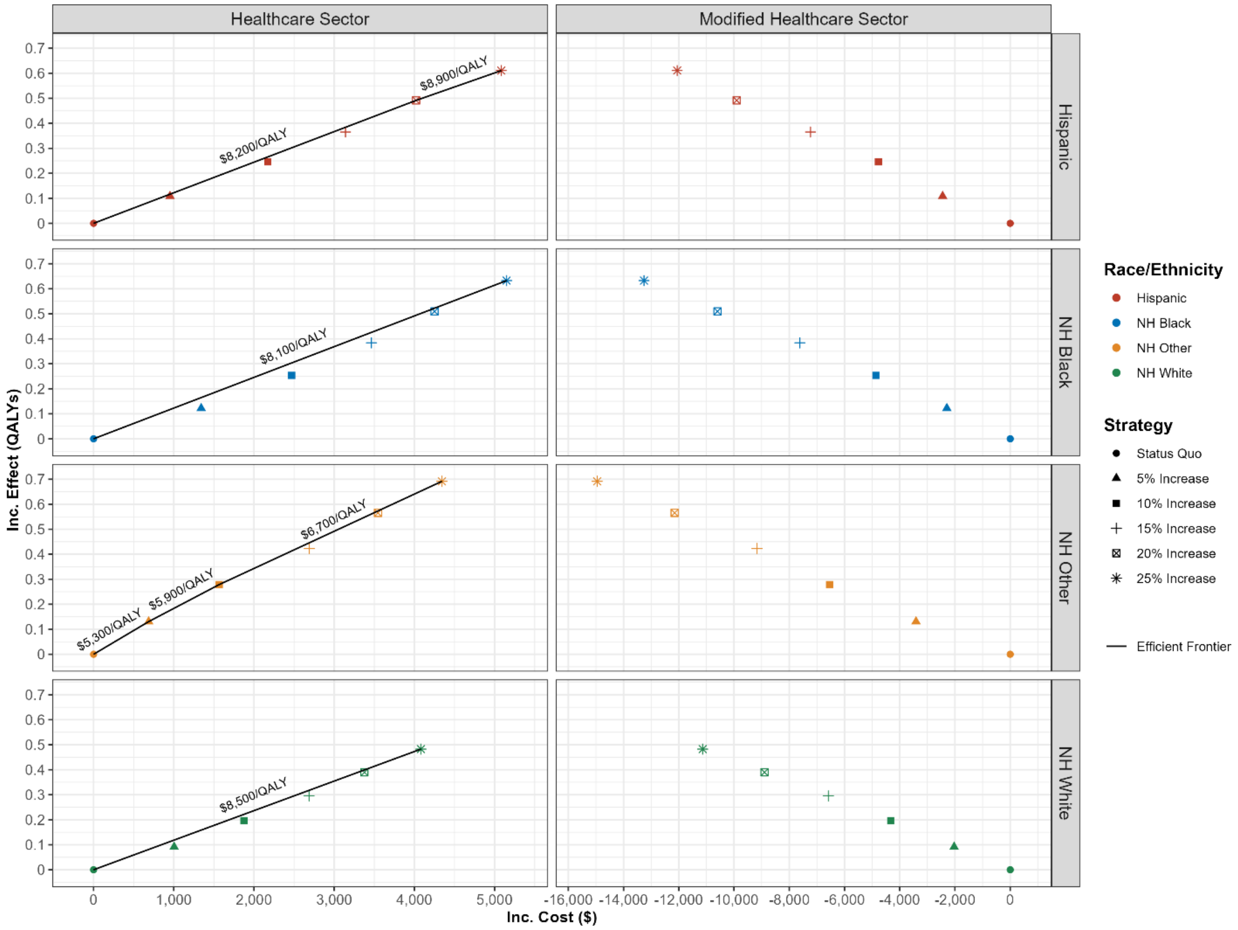

QALYs: quality-adjusted life years
Inc: Incremental

© 2026 Kaufmann MB et al. *JAMA Network Open.*

eFigure 11. Incremental Cost-Effectiveness Frontier by Diabetes History and Perspective

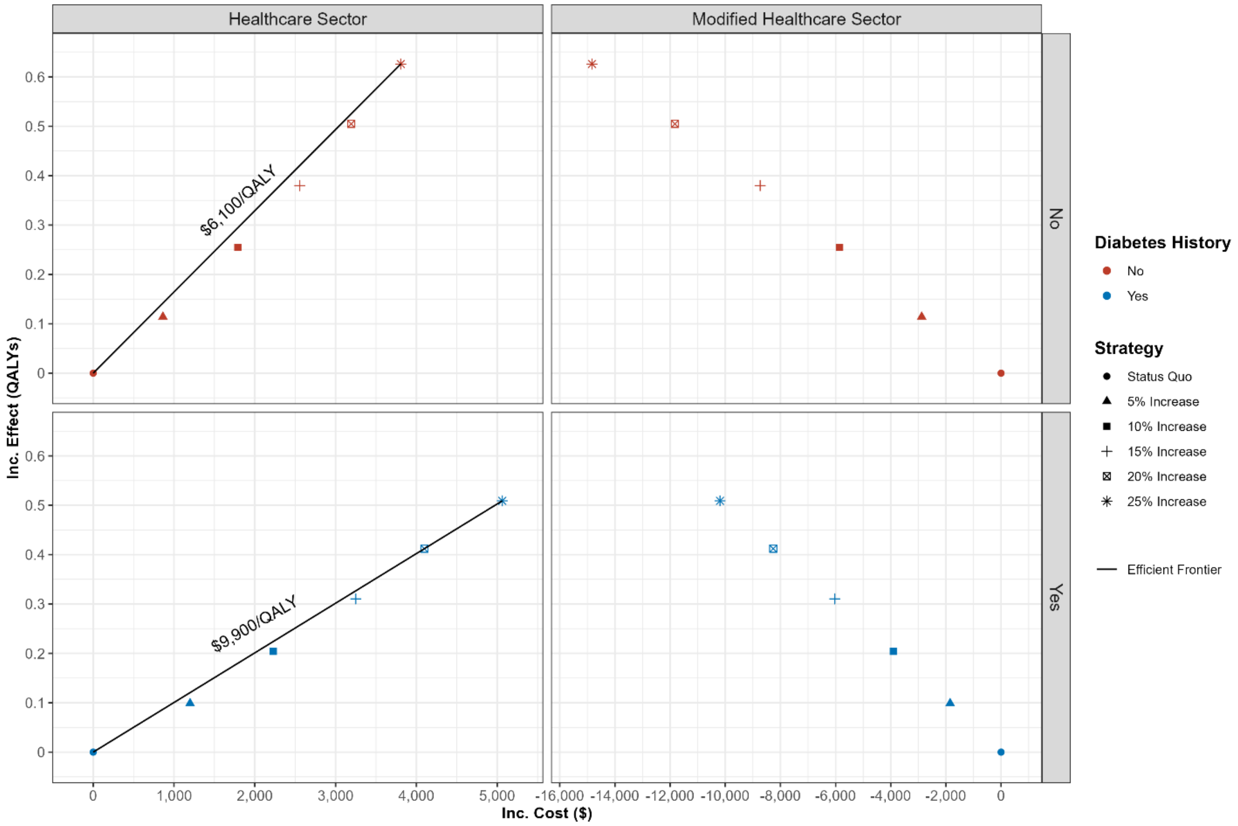

QALYs: quality-adjusted life years

Inc: Incremental

© 2026 Kaufmann MB et al. *JAMA Network Open.*

eFigure 12. Incremental Cost-Effectiveness Frontier by Perspective and the Percentage of Kidneys With Worse Quality Than Their KDPI Implies

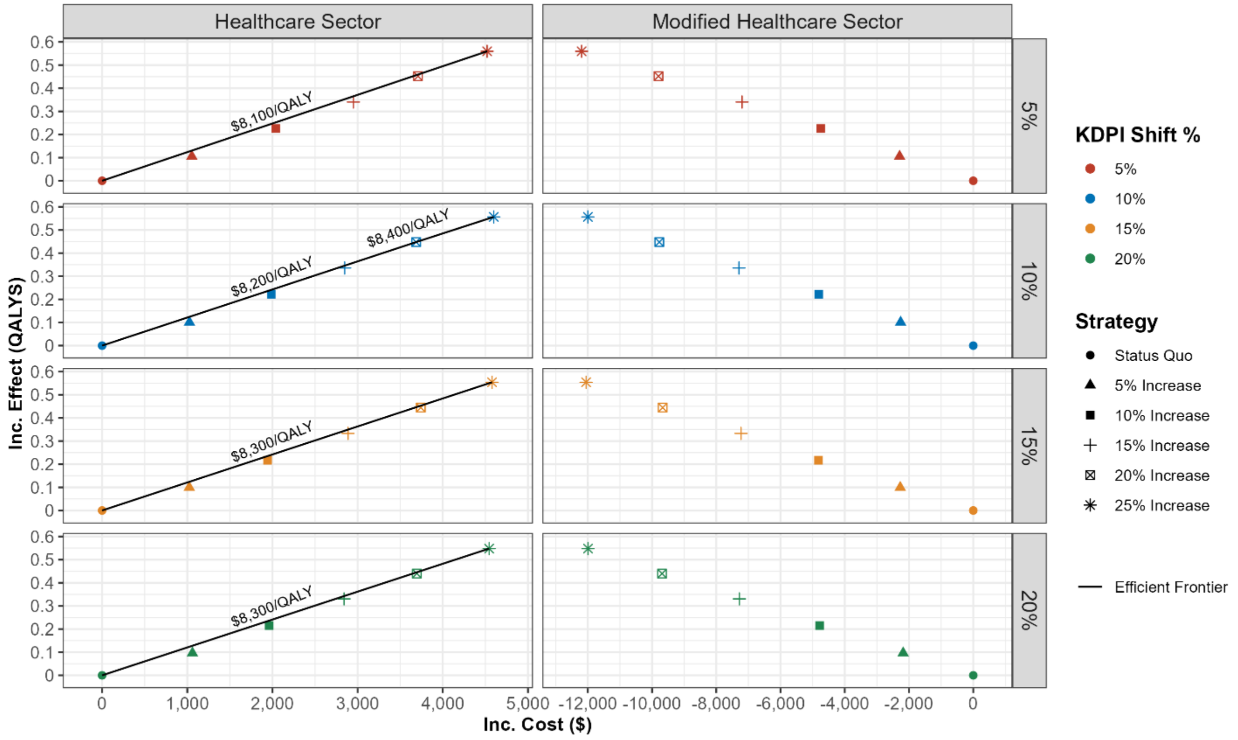

QALYs: quality-adjusted life years
Inc: Incremental
KDPI: kidney donor profile index

© 2026 Kaufmann MB et al. *JAMA Network Open.*

eFigure 13. Expected Loss Curves by Perspective

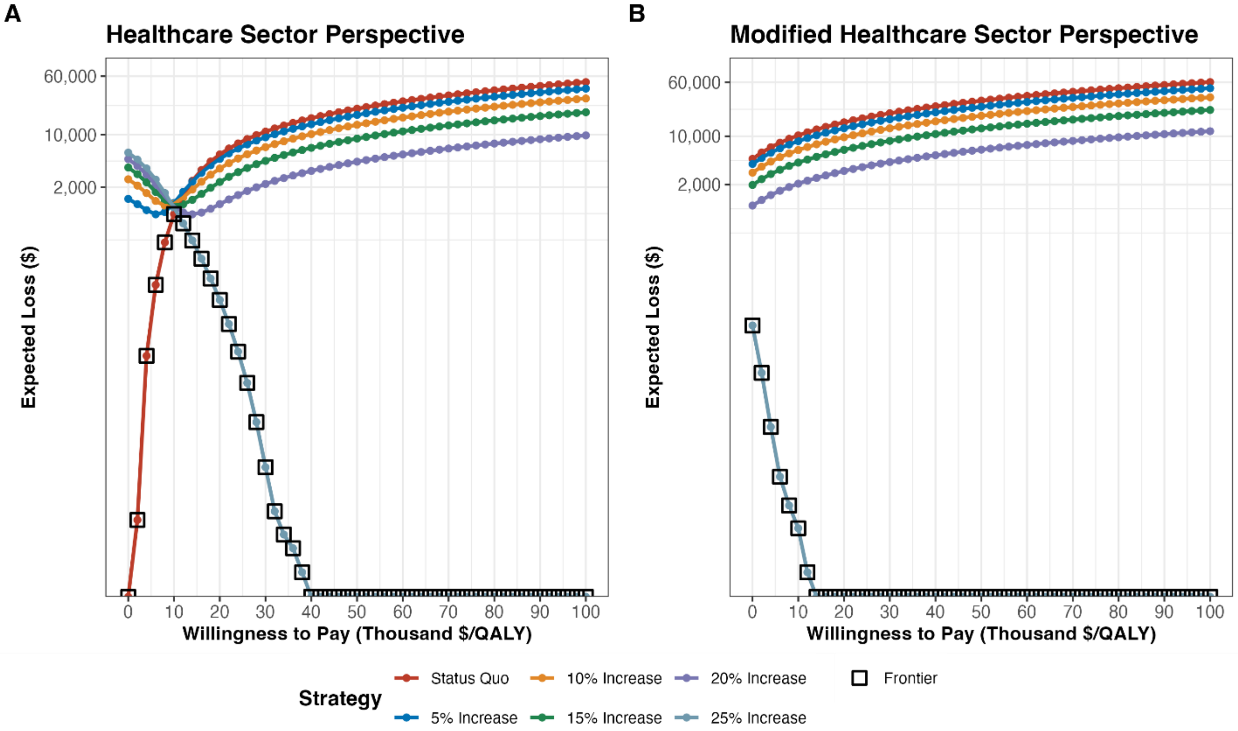

Expected loss curves from A) healthcare sector perspective, and B) modified healthcare sector perspective.
QALY: quality-adjusted life year

© 2026 Kaufmann MB et al. *JAMA Network Open.*

eReferences.

1. *2023 USRDS Annual Data Report: Epidemiology of Kidney Disease in the United States*. https://adr.usrds.org/2023

2. Almond PS, Matas AJ, Canafax DM. Fixed-Rate Reimbursement Fails to Cover Costs for Patients with Delayed Graft Function. *Pharmacotherapy: The Journal of Human Pharmacology and Drug Therapy*. 1991;11(5):126S-129S. doi:10.1002/j.1875-9114.1991.tb02642.x

3. U.S. Bureau of Economic Analysis, Personal consumption expenditures: Services: Health care [DHLCRC1Q027SBEA]. Retrieved from FRED, Federal Reserve Bank of St. Louis. February 14, 2024. https://fred.stlouisfed.org/series/DHLCRC1Q027SBEA

4. Axelrod DA, Schnitzler MA, Xiao H, et al. The Changing Financial Landscape of Renal Transplant Practice: A National Cohort Analysis. *American Journal of Transplantation*. 2017;17(2):377-389. doi:https://doi.org/10.1111/ajt.14018

5. *2021 USRDS Annual Data Report: Epidemiology of Kidney Disease in the United States*. https://adr.usrds.org/2021

6. HCUPnet Data Tools – Healthcare Cost and Utilization Project (HCUPnet). Accessed September 3, 2024. https://datatools.ahrq.gov/hcupnet/

7. Hanmer J, Lawrence WF, Anderson JP, Kaplan RM, Fryback DG. Report of Nationally Representative Values for the Noninstitutionalized US Adult Population for 7 Health-Related Quality-of-Life Scores. *Med Decis Making*. 2006;26(4):391-400. doi:10.1177/0272989X06290497

© 2026 Kaufmann MB et al. *JAMA Network Open.*

8. Wyld M, Morton RL, Hayen A, Howard K, Webster AC. A Systematic Review and Meta-Analysis of Utility-Based Quality of Life in Chronic Kidney Disease Treatments. *PLOS Medicine*. 2012;9(9):e1001307. doi:10.1371/journal.pmed.1001307

9. Hanmer J, Lawrence WF, Anderson JP, Kaplan RM, Fryback DG. Report of Nationally Representative Values for the Noninstitutionalized US Adult Population for 7 Health-Related Quality-of-Life Scores. *Med Decis Making*. 2006;26(4):391-400. doi:10.1177/0272989X06290497

10. Kaufmann MB, Tan JC, Chertow GM, Goldhaber-Fiebert JD. Deceased donor kidney transplantation for older transplant candidates – a new microsimulation model for determining risks and benefits. *Med Decis Making*. 2023;43(5):576-586. doi:10.1177/0272989X231172169

11. Stinnett AA, Paltiel AD. Estimating CE Ratios under Second-order Uncertainty: The Mean Ratio versus the Ratio of Means. *Med Decis Making*. 1997;17(4):483-489. doi:10.1177/0272989X9701700414

12. Kuntz KM, Weinstein MC. Modelling in economic evaluation. In: Drummond M, McGuire A, eds. *Economic Evaluation in Health Care: Merging Theory with Practice*. Oxford University Press; 2001:0. doi:10.1093/oso/9780192631770.003.0007

© 2026 Kaufmann MB et al. *JAMA Network Open.*
