# Supplementary material for: Cost-Effectiveness of Acceptable-Quality Deceased Donor Kidneys for Transplant in Older Candidates
Source: JAMA Netw Open. 2026 Jan 27;9(1):e2555428. doi: 10.1001/jamanetworkopen.2025.55428 (PMC12848627; doi:10.1001/jamanetworkopen.2025.55428)
Supplement: Supplement 2. — Data Sharing Statement [file jamanetwopen-e2555428-s002.pdf]

Data Sharing Statement

Kaufmann. Cost-Effectiveness of Acceptable-Quality Deceased Donor Kidneys for Transplant in Older Candidates. *JAMA Netw Open*. Published January 27, 2026.
doi:10.1001/jamanetworkopen.2025.55428

Data
Data available: Yes
Data types: Data (not involving human participants)
How to access data: https://github.com/mbkauf/OlderKidneyTransplantSim
When available: With publication

Supporting Documents
Document types: Statistical/analytic code
How to access documents: https://github.com/mbkauf/OlderKidneyTransplantSim
When available: With publication

Additional Information
Who can access the data: Data and supporting documents are publicly available via a GitHub repository, available to anyone.
Types of analyses: The data is available for any purpose.
Mechanisms of data availability: The data is available without investigator support.
